# Habitat Predicts Levels of Genetic Admixture in *Saccharomyces cerevisiae*

**DOI:** 10.1534/g3.117.041806

**Published:** 2017-07-10

**Authors:** Viranga Tilakaratna, Douda Bensasson

**Affiliations:** * Faculty of Life Sciences, University of Manchester, M13 9PT, UK; † Department of Plant Biology, University of Georgia, Athens, Georgia 30602; ‡ Institute of Bioinformatics, University of Georgia, Athens, Georgia 30602

**Keywords:** admixture, mosaic, outcrossing, introgression, hybridization, yeast

## Abstract

Genetic admixture can provide material for populations to adapt to local environments, and this process has played a crucial role in the domestication of plants and animals. The model yeast, *Saccharomyces cerevisiae*, has been domesticated multiple times for the production of wine, sake, beer, and bread, but the high rate of admixture between yeast lineages has so far been treated as a complication for population genomic analysis. Here, we make use of the low recombination rate at centromeres to investigate admixture in yeast using a classic Bayesian approach and a locus-by-locus phylogenetic approach. Using both approaches, we find that *S. cerevisiae* from stable oak woodland habitats are less likely to show recent genetic admixture compared with those isolated from transient habitats such as fruits, wine, or human infections. When woodland yeast strains do show recent genetic admixture, the degree of admixture is lower than in strains from other habitats. Furthermore, *S. cerevisiae* populations from oak woodlands are genetically isolated from each other, with only occasional migration between woodlands and local fruit habitats. Application of the phylogenetic approach suggests that there is a previously undetected population in North Africa that is the closest outgroup to the European *S. cerevisiae*, including the domesticated Wine population. Careful testing for admixture in *S. cerevisiae* leads to a better understanding of the underlying population structure of the species and will be important for understanding the selective processes underlying domestication in this economically important species.

The wine yeast *Saccharomyces cerevisiae* is one of the most economically important model organisms, and is used by humans around the world to produce alcohol and to ferment foods (Fay and Benavides 2005; Wang *et al.* 2012; Gallone *et al.* 2016; Gonçalves *et al.* 2016). *S. cerevisiae* is also found in the wild on fruits, flowers, on the bark of trees in oak woodlands, and can occur as a commensal or pathogen of humans (Sniegowski *et al.* 2002; Wang *et al.* 2012; Hyma and Fay 2013; Goddard *et al.* 2010; Cromie *et al.* 2013; Dashko *et al.* 2016; Goddard and Greig 2015). *S. cerevisiae* shares the oak woodland habitat with all other *Saccharomyces* species, suggesting that the woodland habitat is ancestral for the species (Eberlein *et al.* 2015). The presence of *S. cerevisiae* on a broad range of habitats compared to its closest relatives makes it an ideal model for molecular ecology, especially because many genome-wide technologies are developed and tested first on *S. cerevisiae*, providing a wealth of supporting resources to help interpret ecological patterns (Cherry *et al.* 2011).

However, the presence of genetic admixture among natural strains of yeast presents a challenge for the use of *S. cerevisiae* as a model in population genomics (Liti *et al.* 2009; Almeida *et al.* 2015; Ludlow *et al.* 2016; Barbosa *et al.* 2016). Indeed, genetic admixture also complicates the population genomic analysis of model plants (Hufford *et al.* 2013; Brandvain *et al.* 2014) and animals (Pool *et al.* 2012), including humans (Sankararaman *et al.* 2014; Harris and Nielsen 2016). In addition to informing population genomic analysis, the study of genetic admixture can also reveal signatures of selective processes in natural (Brandvain *et al.* 2014) and human commensal populations (Hufford *et al.* 2013; Pool *et al.* 2012). Introgressions from natural to domesticated populations can allow adaptation of crops to local habitats (Hufford *et al.* 2013) and has probably played an important role in animal domestication (Marshall *et al.* 2014). The study of genetic admixture with deleterious effects in natural populations also has potential applications in conservation biology (Harris and Nielsen 2016). In the case of *S. cerevisiae*, analysis of genetic admixture could potentially reveal mechanisms of adaptation to industrial applications and the human body, as well as the connectivity of natural populations within and between habitats.

Past studies have employed a number of different approaches to test whether strains are “mosaics” (genetically admixed), which precludes comparison among studies or samples (Liti *et al.* 2009; Wang *et al.* 2012; Cromie *et al.* 2013; Almeida *et al.* 2015; Barbosa *et al.* 2016). The precise definitions differ among studies, but in general, admixed *S. cerevisiae* strains are identified as (i) those that have long branches in phylogenetic analyses and do not occur in well-supported clades with other strains (Liti *et al.* 2009; Wang *et al.* 2012), or (ii) those that are not assigned to distinct populations using a Bayesian clustering method (Liti *et al.* 2009; Almeida *et al.* 2015; Wang *et al.* 2012; Cromie *et al.* 2013; Barbosa *et al.* 2016). In all cases, conclusions are based on analysis of data from multiple loci concatenated into a single alignment. One drawback of both the phylogenetic and Bayesian approaches as they are usually implemented is that strains belonging to populations that are poorly represented in a sample may incorrectly be defined as mosaics.

Here, we test for differences in the levels of admixture among *S. cerevisiae* found in different habitats using two different approaches. We employ the most commonly used Bayesian test to detect admixture in our study strains (Pritchard *et al.* 2000). In addition, we develop a locus-by-locus phylogenetic approach in which a strain only tests positive for admixture when its different loci are assigned with good statistical support to differing populations. We avoid the complicating effects of selection and recombination in our analysis by using sequences for the point centromeres of *S. cerevisiae*, which are easy to sequence, are neutrally and rapidly evolving, and have low recombination rates (Bensasson *et al.* 2008). For our sampling strategy, we focus on woodland and fruit habitats in proximity to each other and compare these naturally occurring strains to a worldwide panel of *S. cerevisiae* (Table 1). Using complete DNA sequence for the centromeres and the flanking DNA of all 16 chromosomes from 80 *S. cerevisiae* strains, we show differences between habitats in levels of genetic admixture and that oak woodland populations are more isolated than those from other habitats.

**Table 1:** Summary of the 80 *S. cerevisiae* strains used in this study

Geographic Region	Geographic Origin	Habitat	Number of Strains	Study
USA	North Carolina	Oaks	14	Diezmann and Dietrich (2009)
		Grapes	10	Diezmann and Dietrich (2009)*^a^*
	Pennsylvania	Oaks	10	Sniegowski *et al.* (2002)
Europe	Portugal, Greece, and Hungary	Oaks	12	Sampaio and Gonçalves (2008), Robinson *et al.* (2016), Almeida *et al.* (2015)*^b^*
	Greece	Figs	3	Robinson *et al.* (2016)
Worldwide	Multiple*^c^*	Multiple	31	Liti *et al.* (2009)

a Strains were collected by Anne Rouse and kindly provided by Greg Wray.

b A strain from Hungary was kindly provided by Eladio Barrio.

c Further details for this sample and all other strains are in File S1.

## Materials and Methods

### Yeast strains and DNA sequencing

We analyzed DNA sequence data from all 16 centromeres for 80 *S. cerevisiae* strains (Supplemental Material, File S1 and Table 1). Centromere sequences were already available for 33 strains (Table 1; Bensasson 2011; GenBank: HQ339369–HQ339877), and we reuse these data here. These previously-reported sequences were obtained from monosporic derivatives, which we expect to be completely homozygous in all parts of the genome except at the MAT locus (Bensasson 2011).

For the remaining 47 strains, we generated monosporic derivatives by sporulating yeast and isolating single spores as described in Amberg *et al.* (2005). DNA was extracted, amplified, and sequenced from the monosporic derivatives using the extraction, PCR, and DNA sequencing conditions described in Bensasson (2011). DNA sequence reads were assembled into a consensus DNA sequence for each strain at each locus using Staden version 1.7.0 (Bonfield *et al.* 1995) and its quality was assessed using Phred (version: 0.020425.c) as described in Bensasson (2011). Low-quality bases at the ends of consensus sequences were trimmed and any other bases with a Phred-scaled quality score below q40 were masked. The methods used here and the q40 filter ensure a very low base-calling error rate (Bensasson 2011). The resulting centromere sequences are available in GenBank (KT206234–KT206982). Sequences were manually aligned and visualized in SeaView 4.0 (Gouy *et al.* 2010).

### Phylogenetic analysis

Alignments for all 16 centromere loci were concatenated into a single long alignment using a custom perl script (alcat.pl). Genetic distances between DNA sequences were estimated and analyzed using the ape package (version 3.5 Paradis 2011) in R (version 3.3.0). More specifically, we estimated genetic distance using the F84 model (Felsenstein and Churchill 1996) as implemented in dist.dna and constructed a neighbor joining tree (Saitou and Nei 1987) from these distances using nj (Paradis 2011). DNA sequence data were bootstrapped using boot.phylo with 10,000 replicates to test the statistical support of the clades obtained. The resulting phylogram with associated bootstrap values was visualized and colored using plot.phylo.

A second phylogenetic analysis was performed, after excluding strains showing recent genetic admixture (File S1). We conducted a phylogenetic analysis of the concatenated alignment of data for all loci using the maximum likelihood approach implemented in RAxML (version 8.2.4; Stamatakis 2014). We used a general time reversible model with a γ distribution to estimate rate heterogeneity at sites from the data (GTRGAMMA), and a rapid bootstrap analysis for 10,000 bootstrap replicates with a search for the best-scoring Maximum Likelihood tree in the same RAxML run.

### Population structure analysis

We tested for population structure within our sample of 80 yeast strains using the software package *structure* (version 2.3.4) with a model taking account of linkage between polymorphisms at the same locus (Falush *et al.* 2003), and assuming that the DNA sequences were haploid. Using *structure*, we estimated the most likely number of populations to explain the data (K) by varying K from 1 to 10 and visualizing the results. The linkage model allows individuals to show admixture between the K different populations (INFERALPHA 1), and we ran it with default parameters: a burnin of 10,000 steps followed by a run length of 20,000 steps. In pilot experiments, we found that increasing the length of the burnin from 10,000 to 100,000 did not alter our conclusions. Runs were repeated for each value of K five times. There are multiple methods for deciding the value of K that best describes the data (Pritchard *et al.* 2000; Evanno *et al.* 2005; Hubisz *et al.* 2009); here, we use the method recommended by Pritchard and others in the original *structure* publication (Pritchard *et al.* 2000) and more recently in Hubisz *et al.* (2009). More specifically, we identified the most likely models across the 50 runs and from these we selected the model with the lowest value of K (Pritchard *et al.* 2000; Hubisz *et al.* 2009). We checked that all the models that were similarly likely had higher values of *K*. In addition, we verified that the most likely models with larger values of K resulted in groupings of strains with respect to their sampling location that are similar to the best model discussed in the *Results*.

**Figure 1:**
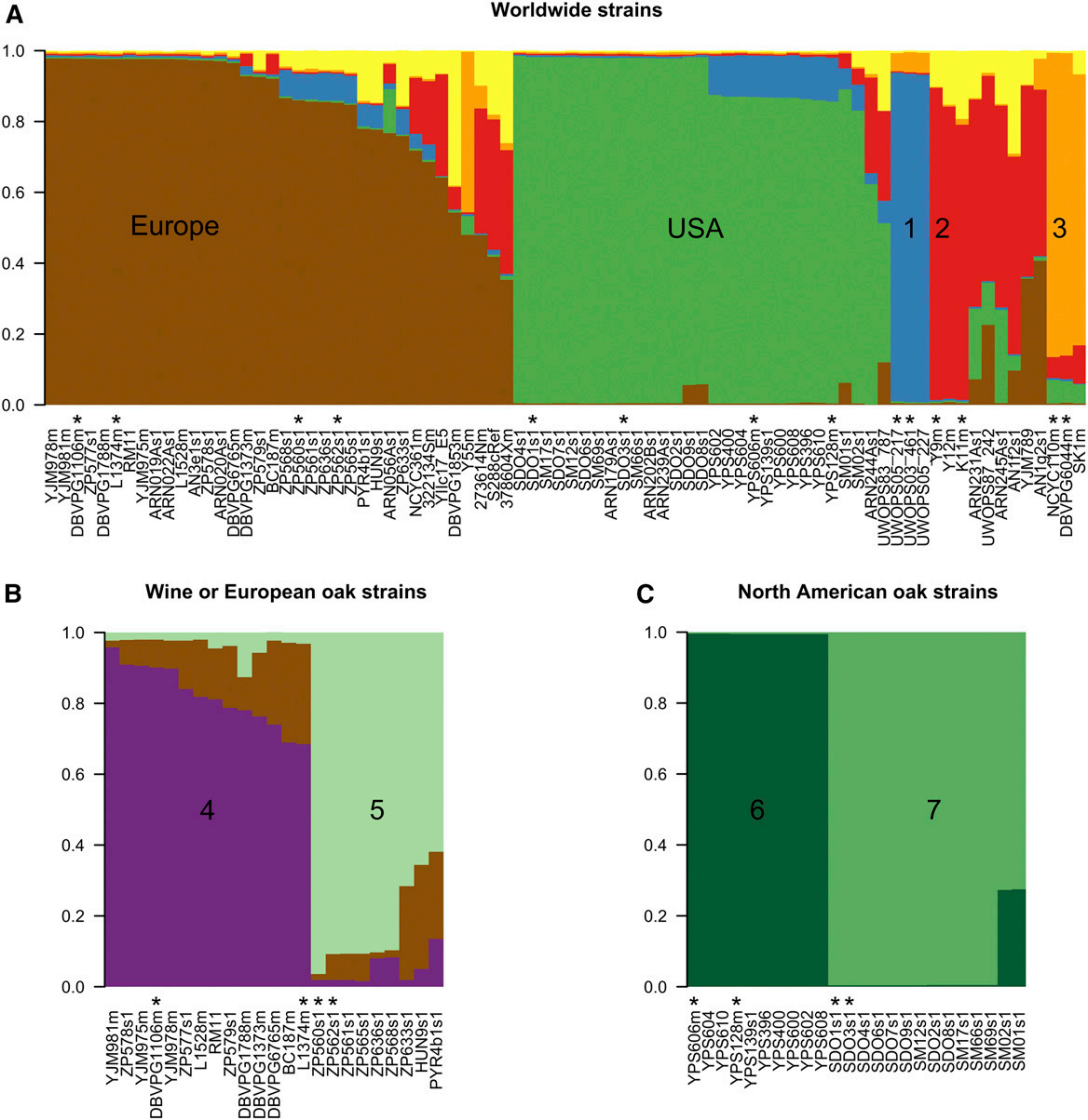
Identification of seven *S. cerevisiae* populations when including the subpopulations within Europe and the USA. A breakdown of population assignments defined by the most likely models estimated by *structure* analysis. Reference strains for the detection of admixture in subsequent analyses are highlighted with a “*”. (a) Five major global populations identified in the worldwide sample. Most of our worldwide sample of 80 strains can be assigned to five distinct populations: “1” is the Malaysia population (blue), “2” is the population from Sake (red), and “3” is the West Africa population (orange), as well as strains from Europe (brown) and the USA (green). *structure* also invoked some gene flow from a sixth (*K* = 6) population (yellow); however, we did not encounter any strains that clearly represent this population. (b) Two subpopulations identified in Europe. Analysis of the 23 strains that were isolated from European oak trees or were previously assigned to a European Wine population (Liti *et al.* 2009) shows that there are at least two *S. cerevisiae* subpopulations in Europe (K = 3): population “4” from wine (purple) and population “5” from European Oaks (green). (c) Two subpopulations identified in the USA. Analysis of the 24 strains isolated from oaks in the USA shows that there are two *S. cerevisiae* subpopulations in the USA (K = 2): population “6” from Pennsylvanian Oaks (dark green) and population “7” from North Carolina Oaks (light green).

The *structure* package was run using three perl scripts available at https://github.com/bensassonlab/scripts: (i) structureInfile.pl converts sequence alignments (in fasta format) to *structure* input files that summarize bases at variable sites; (ii) structureShell.pl runs *structure* one time for each value of K in a specified range (from 1 to 10 in this study); and (iii) structurePrint.pl plots the structure results as barplots using R and allows for user specification of colors (Figure 1).

**Figure 2:**
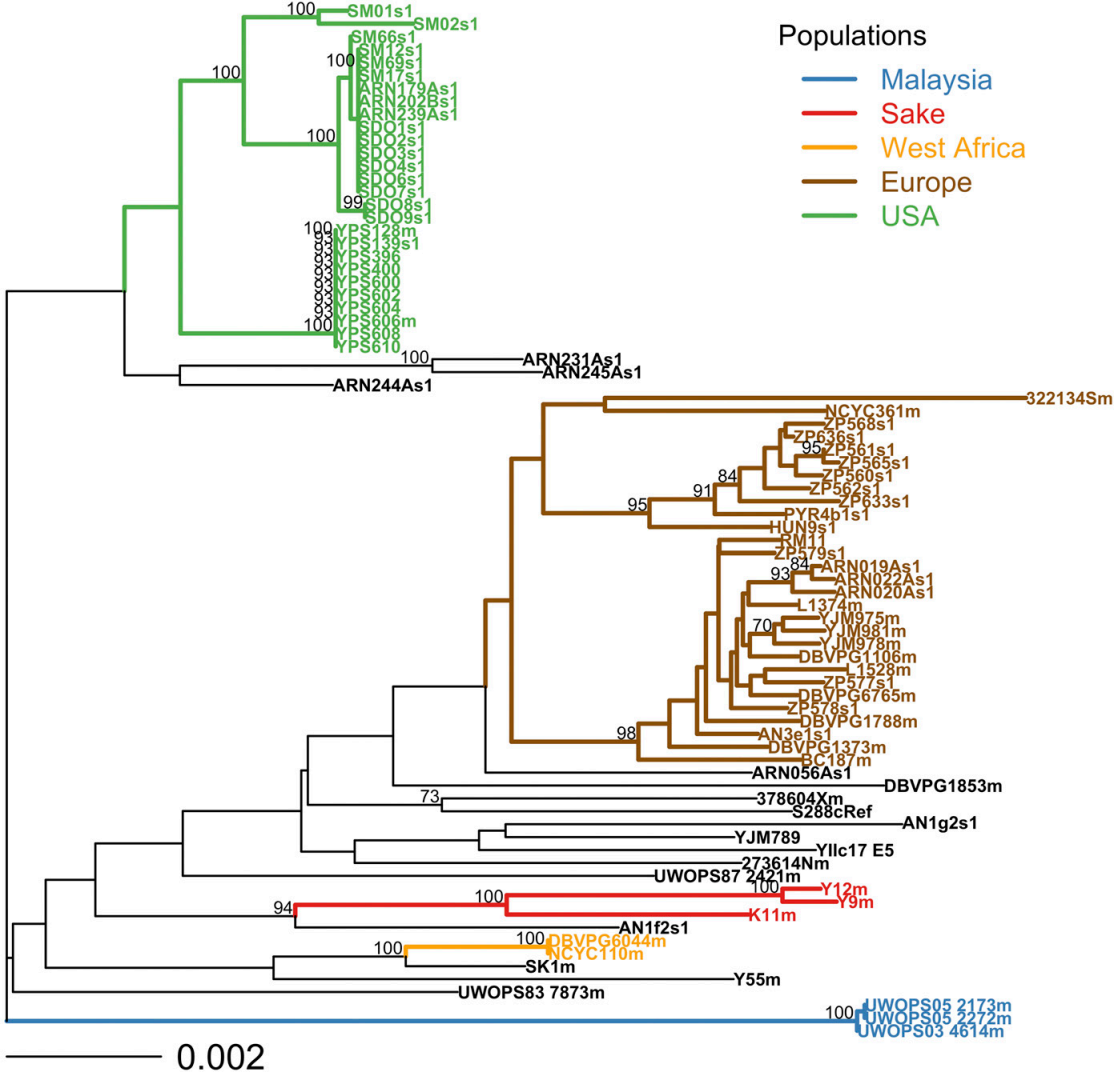
Neighbor joining distance analysis of all strains shows subpopulations within the USA and Europe. Bootstrap support was estimated from 10,000 bootstrap replicates and support is shown as a percentage for clades with over 70% support. Strain names and clades are colored according to populations defined by the *structure* analysis shown in Figure 1A. Bootstrap support for the European and USA populations is < 70%; however, there is strong bootstrap support (≥ 95%) for subpopulations within the USA and Europe.

Our preliminary phylogenetic analysis showed well-supported subpopulations within Europe and the USA (at least 95% bootstrap support for all four subpopulations in Figure 2) that are missed by the initial *structure* analysis (Figure 1a). Following the recommendations in the *structure* documentation for identifying subpopulations, we repeated the above *structure* analysis of 50 runs (K = 1 to 10, 5 replicates) on two subsets of the data: (i) on the 23 strains that were either assigned to the European Wine population by Liti *et al.* (2009) or were isolated from European oak trees (Figure 1b and File S1); and (ii) on the 24 strains isolated from oaks in the USA (Figure 1c and Table 1). The worldwide sample of strains used here (Table 1) and by others (Liti *et al.* 2009) includes too few strains in the remaining three populations to permit testing for further subpopulations. Liti *et al.* (2009) assigned only three strains to the “Malaysia” population, three strains to the “Sake” population, and two strains to the “West Africa” population, and our analysis of the same worldwide sample of strains supports these population designations (Figure 1 and Figure 2).

In order to test whether the *structure* analyses make meaningful population predictions, we used ObStruct (version 1.0; Gayevskiy *et al.* 2014). ObStruct tests whether ancestry estimates (Figure 1) were correlated with the population assignments that we expect based on the geographic locations shown in Table 1 or on past assignment to the European Wine population (Figure 1c; Liti *et al.* 2009). We were able to apply ObStruct to the best *structure* models because no sampling location information was used in generating them (Figure 1). For our ObStruct analysis of the initial model generated by *structure* (Figure 1a), we included only our 49 study strains and excluded the worldwide panel of 31 strains, many of which had uncertain sampling locations (Table 1).

### Testing for recent genetic admixture

In order to test for genetic admixture between populations, we first defined distinct *S. cerevisiae* populations on the basis of *structure* and phylogenetic analyses (Figure 1 and Figure 2). From each of the seven different populations identified in this way, we chose two reference strains to define each population (the minimum number that *structure* needs to estimate allele frequencies within a population). Where possible, reference strains were chosen from strains that were assigned to a particular population in an independent study (Liti *et al.* 2009). Liti *et al.* (2009) assigned more than two strains to the Malaysian, Sake, and Wine populations, and in these cases, for our reference strains we selected strains isolated from the habitat described by the population name: strains isolated from wine for the Wine population or from sake for the Sake population, and in cases where more than two strains fit these criteria, we selected the strains with the highest estimates of genetic ancestry for their population. For example, for the Wine population, we chose strains DBVPG1106m and L1374m, which were previously assigned to the Wine population by Liti *et al.* (2009) and were isolated from wine. For the populations that were not identified by Liti *et al.* (2009), European Oak and North Carolina Oak, reference strains were those with the highest estimates of genetic ancestry for their respective population (Figure 1, b and c).

After removing the 14 reference strains, the remaining 66 strains in this study were assigned to populations or defined as showing recent genetic admixture using two independent approaches. The first approach uses *structure* to estimate levels of admixture for every strain with reference strains to define distinct populations using the USEPOPINFO option (Pritchard *et al.* 2000). It was necessary to define populations in this way because the initial runs of *structure* missed the subpopulations within Europe and North America. Preliminary analysis of the results of our initial 50 replicate runs of *structure* suggested that, in cases where population substructure was missed (European Oak and North Carolina Oak populations), the admixture invoked to explain the divergence of these populations from the rest was inconsistent. For example, in different replicate runs of *structure*, admixture from a single other population was invoked to explain the divergence of European Oak strains from the Wine population, but the source of the admixture was inconsistent: sometimes Malaysia, Sake, or West Africa. From this, we concluded that admixture is sometimes invoked by *structure* to explain divergence between populations. Therefore, we developed a second locus-by-locus phylogenetic test for admixture that only classifies strains as admixed if they show well-supported similarity to multiple populations, and are thus more conservative. Details of both approaches are described in the following sections.

#### Detecting admixture using structure:

We ran *structure* using the same parameters as we used for defining populations above, except that we used the USEPOPINFO model in *structure* (Pritchard *et al.* 2000) to estimate ancestry for the 66 *S. cerevisiae* strains of unknown origin (POPFLAG = 0) by prespecifying the population of origin for the 14 reference strains (POPFLAG = 1). We set the number of populations to seven (K = 7), and selected the breakdown of ancestry components for each strain from the most likely model out of 20 independent runs. For consistency across our two approaches, we defined a strain as “admixed” if its proportion of ancestry to a single population was < 0.94 (equivalent to 1 out of 16 centromere loci being from a different population).

#### Detecting admixture using a locus-by-locus phylogenetic approach:

We also performed locus-by-locus phylogenetic analyses of all 80 strains, assigning each of the 66 nonreference strains at each locus to a population according to which of the 14 reference strains it grouped with. We used custom perl scripts to run the phylogenetic analysis using the ape package in R (chrbychr.pl and chrbychr2.pl). For each locus, we constructed a neighbor joining tree of genetic distances using the F84 model, used 10,000 bootstrap replicates to assess statistical support for each clade, and output a text summary of the strains found in each clade using the prop.part tool of boot.phylo in ape (Paradis 2011). We only considered clades that had at least 70% bootstrap support. For each locus, strains found in clades with reference strains from only one population were assigned to the same population as the reference strains. Because of the limited phylogenetic resolution at some loci, we also assigned strains in clades with reference strains only from the European Oak or Wine populations as “European” and those in clades with North Carolina or Pennsylvania reference strains as “USA.” In cases where a sequence does not group with those of reference strains belonging to a single population, its population status at that locus is classed as “undefined.” The more general classifications of European or USA do not conflict with subpopulation classifications within those groups, and loci classed as undefined do not conflict with classifications at any other loci. We then compared population predictions across all loci for a strain, and if a strain was assigned to a population at a locus that conflicted with the population assignment at any of the other loci, then that strain was defined as showing genetic admixture. For example, SDO8s1 was isolated from a North Carolina oak, and has a CEN4 sequence from the Wine clade, but it has five loci that are in the same clade as the North Carolina Oak reference strains (the remaining loci were undefined or in the USA clade; Figure S1). Therefore, this strain shows admixture between Wine and North Carolina Oak populations.

In most cases (55 out of 66 strains), the two approaches resulted in the same population and admixture assignments. Fewer strains were defined as admixed using the locus-by-locus phylogenetic approach (Table 2), because a locus is classed as undefined when there is insufficient data at a locus for population assignment. Therefore, we used this more conservative approach to decide which strains to exclude from the final phylogenetic analysis.

**Table 2:** *S. cerevisiae* from trees in oak woodlands show less genetic admixture than those from other habitats

	*structure* Analysis			Phylogenetic Analysis		
Habitat	Not Admixed	Admixed	Proportion Admixed	Not Admixed	Admixed	Proportion Admixed
Fruit or flower	9	12	0.57	11	10	0.48
Human infections	3	4	0.57	3	4	0.57
Fermentations	7	5	0.42	9	3	0.25
Soil and unknown	0	4	1.00	3	1	0.25
Oak or other trees	32	4	0.11*^a^*	32	4	0.11*^a^*

a Fisher’s exact tests show that habitats differ in the prevalence of strains that show recent admixture (*structure* analysis: $P=4\times 10^{-5};$ locus-by-locus analysis: P=0.009). If strains isolated from oaks or other trees are excluded, then the prevalence of strains with recent admixture is independent of habitat (*structure* analysis: P=0.3; locus-by-locus analysis: P=0.5), suggesting that most of the difference among habitats is due to the low genetic admixture seen in woodland strains.

As an experimental control to test whether the number and choice of reference strains might affect our results and conclusions, we also tested for recent genetic admixture using both the *structure* and the locus-by-locus phylogenetic approach, but with a much longer list of 38 reference strains. These 38 strains included all the strains assigned to five populations in an independent study (Liti *et al.* 2009), and the strains assigned with the highest confidence (ancestry >0.9) to the North Carolina or European Oak subpopulations. Our findings and conclusions were unchanged in these control runs, except that we necessarily missed cases of genetic admixture in strains that were included in this larger set of reference strains. In addition, we tested the effect of simply using the two strains with the highest estimates of genetic ancestry for each population as reference strains. Using the locus-by-locus phylogenetic approach, this resulted in a different classification for only one strain (K11 was classed as admixed and not a Sake strain), and designations for four strains were affected by the choice of reference strains with the *structure* approach. In all cases, there were only negligible effects on the proportions of strains that were admixed from different habitats, and none of our findings or conclusions were affected. We did not use these control analyses further.

### Data availability

DNA sequences determined for this study are available in GenBank: KT206234–KT206982. Perl scripts are available at https://github.com/bensassonlab/scripts, and locus-by-locus alignments and tree file data are available at https://github.com/bensassonlab/data. Most of the yeast strains used in this study are available from the National Collection of Yeast Cultures in the U.K. or the Portuguese Yeast Culture Collection.

## Results

### Seven genetically distinct populations of S. cerevisiae

We generated complete sequence data for whole centromeres from all 16 chromosomes of 47 *S. cerevisiae* strains from oak woodlands and fruit in the USA and Europe. We compared these sequences with a similar dataset previously described in Bensasson (2011) that includes 33 strains collected worldwide (Table 1). The data reused here showed that, in *S. cerevisiae*, centromeres are small (up to 125 bp long), rapidly and neutrally evolving, and have low recombination rates (Bensasson 2011). By analyzing centromeres and their flanking DNA, every yeast chromosome is represented in our analysis. This strategy also minimizes the challenges to phylogenetic and population structure analysis presented by recombination and positive selection (Avise 1994; Posada and Crandall 2002; Brandvain *et al.* 2014) that we would expect in a genome-wide analysis.

Using the Markov Chain Monte Carlo approach implemented in the program *structure* (Pritchard *et al.* 2000; Falush *et al.* 2003), we analyzed data from 895 segregating sites in 13 kb of centromere sequence and identified five main populations in our sample of 80 strains (Figure 1a). Although *structure* used no sampling location information, the assignment of strains to these five populations (ancestry estimates) was highly correlated with geographic sampling location (ObStruct: $R^{2}=0.71,P<0.0001$). These populations also recapitulate those previously described in the worldwide sample of 33 strains: Malaysia, Sake, West Africa, Europe, and the USA (Liti *et al.* 2009). Although these five populations are evident in our phylogenetic analysis of the same dataset, there is not good statistical support for the European and USA clades. However, there do appear to be well-supported distinct subpopulations of *S. cerevisiae* within both Europe and the USA (bootstrap values >95%, Figure 2). These subpopulations are not identified by *structure* in any of the models we obtained, even when invoking a larger number of populations. Therefore, we used a hierarchical approach as recommended in the *structure* documentation to test for population substructure within a subset of European strains (N=23, Figure 1b) and within strains from the USA (N=24, Figure 1c). This analysis revealed two subpopulations within Europe (Wine and European Oak; ObStruct: $R^{2}=0.96,P<0.0001$), and two subpopulations within the USA (Pennsylvania Oak and North Carolina Oak; ObStruct: $R^{2}=0.95,P<0.0001$). Overall, using this hierarchical *structure* approach, we identified a total of seven populations, and these populations were also represented with well-supported clades in our phylogenetic analysis (Figure 2).

### A conservative test for recent genetic admixture

Using the seven populations identified above, we used two approaches to test for genetic admixture in each strain in our dataset. The first approach applies the standard software, *structure*, in a way that is similar to that used by others in the yeast research community to estimate levels of genetic admixture given a panel of reference strains. The initial *structure* analysis that we used to define yeast populations gave inconsistent results invoking admixture from different populations into Europe or North America from one replicate run to the next (see *Materials and Methods*). Therefore, we also developed a second locus-by-locus phylogenetic approach that would only invoke admixture for a strain if it has haplotypes at loci that are assigned with statistical confidence to differing known populations.

**Figure 3:**
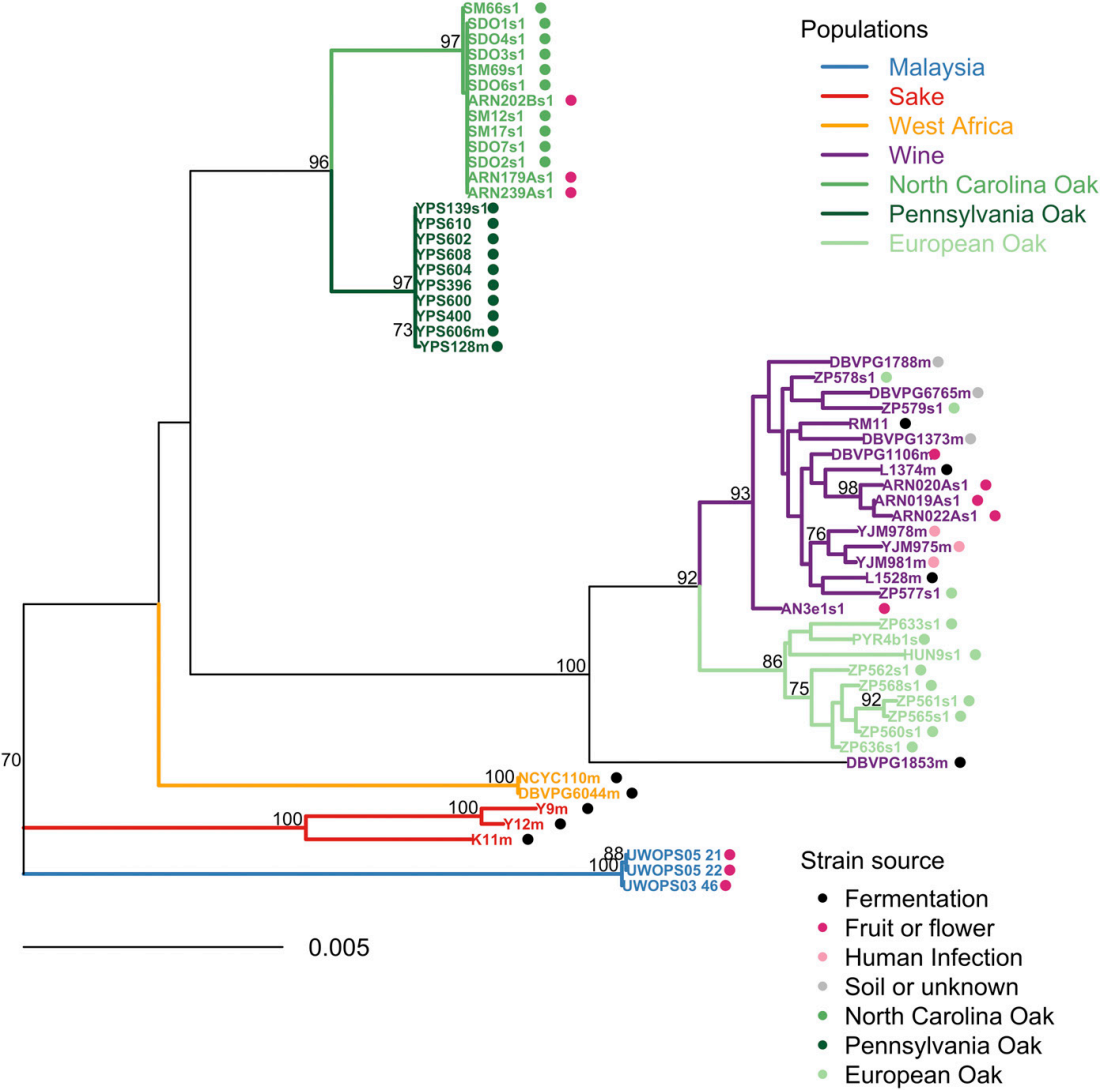
Phylogenetic analysis of nonadmixed strains reveals structured populations with occasional migration. We excluded 22 recently admixed strains out of the 80 strains in the original dataset, and estimated the phylogeny using a maximum likelihood approach with bootstrap support shown for clades with > 70% support out of 10,000 bootstrap replicates. Clades are colored according to their genetic population, and strains are labeled with a colored dot according to their habitat. DBVPG1853m, which is not clearly assigned to a European population in this maximum likelihood analysis, appeared most similar to the Wine population in the locus-by-locus analysis. Strains isolated from oak trees are similar to strains isolated from the same woodland and distinct from those isolated from other regions. There is some migration of *S. cerevisiae* from the North Carolina Oak population onto North Carolina grapes (prefixed “ARN”). In addition, all three strains isolated from oak trees in Aldeia das Dez in Portugal (prefixed “ZP57”) had migrated from the Wine population to trees.

Analysis of admixture results for individual strains showed that 55 out of 66 strains were defined concordantly by the two methods (File S1). In most of the remaining 11 cases, only *structure* invoked admixture in European strains (nine strains, File S1 and File S2). Investigation of all nine strains where only *structure* invokes admixture suggests that these are likely to be false positives because there was no strong or consistent phylogenetic support for their similarity to multiple populations (File S2). For some of these strains, *structure* appeared to invoke admixture to explain genetic divergence from known populations. For example, DBVPG1853m, a strain from North Africa, is most similar to European populations of *S. cerevisiae* yet it is also somewhat diverged from them (Figure 2 and Figure 3). Locus-by-locus analysis showed that it is diverged from European strains, and most similar to the Wine or European populations (File S2 and File S3). For this strain, *structure* invokes admixture between the Wine, European Oak, and Sake populations; however, we see no evidence for greater similarity to European Oak or Sake than other populations at any single locus (File S3).

In other cases, similarity between subpopulations can also lead to the potentially incorrect inference of admixture using the *structure* approach. For example, the wine strain RM11 consistently clustered with Wine or European strains at all loci where there was sufficient data to assign a population using the phylogenetic approach (12 loci, File S2 and File S3). However, *structure* invoked admixture between the European Oak and Wine populations, even though there is no evidence that RM11 is more similar to the European Oak population than the Wine population at any loci (File S3).

Two strains (SDO8s1 and SDO9s1) were defined as admixed using the locus-by-locus phylogenetic approach, but not using the *structure* approach. For both strains, we found strong evidence for admixture from the Wine population to the North Carolina Oak population at a single locus (CEN4, bootstrap support of at least 98%, File S2 and File S3). Using the *structure* approach, we also detected some admixture from Wine to these two North Carolina Oak strains (4.7 and 5.5%), but these levels were below our threshold for defining admixture (one locus out of 16, 6.25%). This analysis of the differences in admixture calls between the two methods suggests that the locus-by-locus phylogenetic method is less likely to call false positives, while still being sensitive enough to detect low levels of admixture within a genome. Therefore, we used the admixture definitions from the locus-by-locus phylogenetic method for subsequent analyses.

### Genetic admixture is high in transient and low in stable habitats of S. cerevisiae

Because *S. cerevisiae* occurs in a broad range of habitats, we can use it as a model organism to test whether there is an effect of habitat on levels of genetic admixture. Fruit, flowers, and insects represent habitats that are transient for yeast (Goddard and Greig 2015; Knight and Goddard 2016) and *S. cerevisiae* also occurs in humans only transiently (Enache-Angoulvant and Hennequin 2005). In contrast, trees are probably undisturbed for decades or even centuries (Knight and Goddard 2016). Therefore, we classified habitats in the worldwide sample of 80 strains according to whether they represent transient natural habitats such as (i) fruit or (ii) human infections, whether their provenance was less well defined from (iii) fermentations, or (iv) soil and unknown sources, or (v) whether they were sampled from well-defined stable woodland habitats (Table 2).

We found that the proportion of admixed strains is lower for strains from oak woodlands (11% of 36 strains) than for those of other habitats (Table 2) using both the *structure* (Fisher’s exact test, $P=4\times 10^{-5}$) and the locus-by-locus phylogenetic approaches (Fisher’s exact test, P=0.009). When we excluded the *S. cerevisiae* from oaks or other trees, the proportion of admixed strains is similar across all habitats (59% of 44 strains, *structure* analysis: P=0.3; 41% of 44 strains, locus-by-locus analysis: P=0.5), suggesting that it is mainly in the woodland habitat that genetic admixture is peculiarly low (Table 2). Therefore, our data suggest that levels of genetic admixture are high in transient and low in stable habitats (Table 2).

Many of the admixed *S. cerevisiae* strains defined using the locus-by-locus phylogenetic method (8 out of 22 strains) showed a complex pattern of admixture involving at least three of the seven defined populations (File S2 and File S3). This suggests that the admixture we see in *S. cerevisiae* is not simply a consequence of recent hybridization resulting in the asexual descendants of F1 individuals. Furthermore, we were able to detect admixture only at a single locus for eight strains, which suggests that in some cases backcrossing has occurred between hybrids and strains from single populations. All four admixed strains from woodlands were isolated from North Carolina Oaks and showed admixture at a single locus, whereas admixture at only single loci occurred less often in strains from other habitats (4 out of 18 strains; Fisher’s exact test, P=0.01). The admixed haplotypes observed in these four North Carolina Oak strains were all identical to (Wine or Malaysian) haplotypes from strains that were isolated from nearby North Carolina grapes. This implies that our results are explained by recent genetic admixture between North Carolina woodlands and local vineyards and not by incomplete lineage sorting. When strains showed admixture at only one locus, the admixture occurred at different loci for nonwoodland strains, but for the four strains from North Carolina Oaks they all showed evidence of admixture at the same locus (CEN4). Indeed, the CEN4 sequence was identical for three of these strains, suggesting that some of this admixture seen in oak strains does not result from independent events, and therefore that we could be overestimating the frequency of admixture in the oak habitat. Overall, the pattern of admixture observed suggests that the degree of admixture, as well as the frequency of admixture, could be lower in oak woodland habitats than in strains from other habitats, and that when admixture does occur it probably originates from nearby yeast strains on fruit.

### Distinct populations and low migration in woodland habitats

The inclusion of DNA sequences that show recent genetic admixture can lead to incorrect phylogenetic estimation, especially when admixture occurred recently between diverged populations (Posada and Crandall 2002), as it does in this study. Thus, to better understand the true relationships between *S. cerevisiae* populations, it is necessary to reduce the effects of recent genetic admixture as much as possible. Therefore, we performed a phylogenetic analysis of the 58 strains in our dataset that did not show recent genetic admixture in our locus-by-locus phylogenetic analysis (Figure 3). Maximum likelihood phylogenetic reconstruction then showed that all but one of these 58 strains can be assigned to the seven known populations. This tree shows none of the long branch mosaic lineages reported in previous genome-wide analysis (Liti *et al.* 2009), suggesting that our admixture filtering was successful.

One strain isolated from white teff grain in North Africa (DBVPG1853m) is not similar to any of the seven study populations. The bootstrap support in the analysis of pooled data from all loci (Figure 3) and in locus-by-locus analyses (File S3) suggests that this strain may be a strain from an undersampled population that represents the closest outgroup to the Wine and European Oak populations of *S. cerevisiae*.

In addition, by overlaying habitat on our tree, phylogenetic analysis in the absence of admixture permits the identification of strains that have potentially migrated between habitats (Figure 3). Yeast strains from different oak woodlands mostly form distinct populations and differ from the strains of other habitats (Figure 3) and we found no evidence for the migration of yeast between oak woodland populations (Figure 3). However, there is evidence for the migration of yeast (i) from the North Carolina Oak population into a local vineyard, (ii) from the Wine population to oak trees, and (iii) from the Wine population to a medley of regions and substrates (Figure 3). Together, these observations suggest that strains from the Wine population migrate more between continents and habitats than the strains with woodland genotypes.

## Discussion

The wine yeast *S. cerevisiae* has tremendous potential as a model for molecular ecology because it occurs naturally in several distinct habitats including fruit, flowers, insects, and the bark of oak trees (Sniegowski *et al.* 2002; Wang *et al.* 2012; Hyma and Fay 2013; Goddard *et al.* 2010; Cromie *et al.* 2013; Dashko *et al.* 2016). *S. cerevisiae* is one of several model organisms whose genomes show evidence of recent genetic admixture from diverged populations (Liti *et al.* 2009; Almeida *et al.* 2015; Ludlow *et al.* 2016; Hufford *et al.* 2013; Brandvain *et al.* 2014; Pool *et al.* 2012; Sankararaman *et al.* 2014). Therefore, *S. cerevisiae* provides an excellent model to test an important question in molecular ecology: whether genetic admixture differs among habitats.

Using two approaches for defining admixed strains in a systematic and quantitative way, we show that patterns of genetic admixture differ between habitats (Table 2). Yeast strains from oak woodlands are less likely to show recent genetic admixture than those from other habitats (Table 2), and when it does occur the degree of admixture is lower and from fewer populations (File S2 and File S3). Consistent with our results, admixture has been noticed in the past for human-associated *S. cerevisiae* strains (Cromie *et al.* 2013; Wang *et al.* 2012) and for those fermenting cacao and coffee (Ludlow *et al.* 2016), but there has been no compelling evidence reported previously for intraspecific admixture in yeast from oak woodlands.

By analyzing neutrally evolving centromeres with low recombination (Bensasson 2011), we minimized the complications of recombination and natural selection on our analysis. Using only these centromere sequences, our results recapitulate genome-wide analyses that identified a second population of *S. cerevisiae* in Europe that represents the closest wild relatives to the *S. cerevisiae* Wine population (Cromie *et al.* 2013; Almeida *et al.* 2015), and the identification of two North American populations by Cromie *et al.* (2013). We were also better able to resolve the North American lineages using only centromere sequences compared to a previous analysis using genome-wide data (Liti *et al.* 2009). Therefore, our analysis of centromere sequences appears to have sufficient power to detect the lineages identified by genomic studies.

We developed a locus-by-locus test to complement the use of *structure*, which is the standard Bayesian method used to estimate admixture (Pritchard *et al.* 2000). A comparison of admixture calls using the two different approaches suggests that *structure* will sometimes invoke admixture to explain the divergence of a strain from defined populations, or it could incorrectly invoke admixture between genetically similar populations (File S2 and File S3). Indeed, by using a locus-by-locus phylogenetic approach in addition to *structure*, we detect evidence for a distinct North African population (represented by DBVPG1853m in Figure 3) that was previously treated as an admixed strain in population genomic analyses (Liti *et al.* 2009; Almeida *et al.* 2015; Cromie *et al.* 2013; Barbosa *et al.* 2016). These findings are consistent with the sensitivity of *structure* and similar programs to sampling scheme when data show isolation by distance (Schwartz and McKelvey 2009). Molecular ecology and population genomic analyses of *S. cerevisiae* have mostly used only *structure* on concatenated alignments from multiple loci to define admixed strains in order to better understand population structure in this species (Wang *et al.* 2012; Almeida *et al.* 2015; Barbosa *et al.* 2016). Our analysis suggests the need for a more thorough investigation of admixture in yeast population genomic data using alternative methods.

When we removed admixed strains from our phylogenetic analyses, it became clear that woodland populations are distinct from one another, even when they occurred relatively close together in the Eastern USA (Figure 3). Given that we were unable to detect any migration (Figure 3) or admixture (File S2) between woodland yeast populations, it seems that yeasts in this ancestral habitat tend to be genetically isolated. Previous reports have suggested distinct oak-associated strains in the primeval forests of China (Wang *et al.* 2012) and Brazil (Barbosa *et al.* 2016). Our findings suggest that genetic isolation is not only a characteristic of Chinese and Brazilian forest populations, but that even strains from trees in Pennsylvania may be genetically isolated from North Carolina tree strains.

Although we do not detect gene flow between oak woodlands, we do find evidence for both migration and admixture between the human-associated Wine population and woodland populations (File S2, File S3, and Table 2). This supports past reports of migration of *S. cerevisiae* between vineyards and local oak trees (Goddard *et al.* 2010; Hyma and Fay 2013). It also mirrors the situation for *D. melanogaster* where human-associated populations show higher admixture than populations from nonurban regions, and there has been recent genetic admixture from cosmopolitan to ancestral populations (Pool *et al.* 2012).

Transient habitats like fruit only exist for a few weeks and therefore must have been colonized recently by yeast (Goddard and Greig 2015; Knight and Goddard 2016). Fruit flies, wasps, bees, and other insects carry live *S. cerevisiae* in their guts and are therefore likely dispersal vectors for the migration of yeast to fruit (Goddard *et al.* 2010; Stefanini *et al.* 2012; Cromie *et al.* 2013; Buser *et al.* 2014). These insects visit multiple fruits and flowers, can fly long distances (Coyne and Milstead 1987; Beekman and Ratnieks 2000), and Drosophilids and honey bees at least have recently expanded to cosmopolitan distributions (Nunney 1996; Whitfield *et al.* 2006; Pool *et al.* 2012). Insects associated with fruit bring together *S. cerevisiae* strains from diverged populations (Stefanini *et al.* 2012). Furthermore, the spores of multiple strains of *S. cerevisiae* can survive passage through the guts of *Drosophila melanogaster* and the survivors are much more likely to undergo mating, and therefore admixture, than uneaten yeasts (Reuter *et al.* 2007). Thus, if yeasts are primarily dispersed by insect vectors, yeasts from transient habitats are more likely to have cosmopolitan distributions and to show recent genetic admixture, as we observe here.

In contrast, oak tree bark is less nutrient rich (Goddard and Greig 2015) and is therefore likely to attract fewer flying insects than rotting fruit. Consistent with this expectation, young oak trees have fewer yeast on their bark than older trees (Robinson *et al.* 2016), suggesting that stable colonization of oak could be occurring over a period of years rather than weeks. Our observation of genetic isolation and low genetic admixture in oak woodland populations is therefore consistent with the lower migration distances and slower colonization expected for oak trees compared to fruit. It is also consistent with the lack of genetic admixture and the isolation by distance seen in *S. paradoxus*, which is the closest relative of *S. cerevisiae* and has been studied almost exclusively from woodlands (Koufopanou *et al.* 2006; Liti *et al.* 2009; Leducq *et al.* 2016). In addition, the degree of divergence that we observe between oak woodland populations may increase with geographic distance even in *S. cerevisiae*: North Carolina oak strains differ from Pennsylvania oak strains, while differing more from European oak strains (Figure 3)

*S. cerevisiae* is especially attractive as a model organism for molecular ecology and population genomics because of the resources already available for understanding its molecular evolution (Kellis *et al.* 2003; Scannell *et al.* 2011), molecular biology (Cherry *et al.* 2011), experimental evolution (Rosenzweig and Sherlock 2014), and for testing predictions in the laboratory (Cubillos *et al.* 2009). Our study shows that the application of better methods for detecting genetic admixture in genomic data from woodland *S. cerevisiae* (Almeida *et al.* 2015; Barbosa *et al.* 2016) could lead to the generation of population genomic data that are unlikely to break the assumptions of most population genetic analyses. Therefore, with more thorough testing for admixture and filtering, *S. cerevisiae* is likely to be an excellent model for population genomic analysis, despite its complex historical association with humans. There is some evidence that introgressions between species could confer adaptive traits in *S. cerevisiae* (Doniger *et al.* 2008) and *S. uvarum* (Almeida *et al.* 2014). Population genomic analysis of intraspecific genetic admixture in maize revealed that gene flow from ancestral populations led to the adaptation of domesticated crops to the Mexican highlands (Hufford *et al.* 2013). Given that *S. cerevisiae* is employed in a broad range of industries, including the production of wine, sake, beer, chocolate, and cacao, it will be especially interesting to apply new tools to study genome-wide patterns of admixture (Corbett-Detig and Nielsen 2017) to reveal whether the genetic admixture seen among populations in *S. cerevisiae* plays a similar adaptive role in domestication.

## Supplementary Material

Supplemental material is available online at http://www.g3journal.org/lookup/suppl/doi:10.1534/g3.117.041806/-/DC1.
