# Supplementary material for: Habitat Predicts Levels of Genetic Admixture in Saccharomyces cerevisiae
Source: G3 (Bethesda). 2017 Jul 10;7(9):2919–29. doi: 10.1534/g3.117.041806 (PMC5592920; doi:10.1534/g3.117.041806)
Supplement: Supplementary file 1 [file 2919FileS1.pdf]

Table S1 : Sources of *Saccharomcyes cerevisiae* strains and their genotypes.

Name	Strain Source[a]	Geographic Origin	Habitat Origin	Habitat Category[b]	*structure* lineage[c]	Locus-by-locus lineage[c]
273614Nm	NCYC	Royal Victoria Infirmary, Newcastle, UK	Clinical isolate (Fecal)	Human infections	Admixed	Admixed
322134Sm	NCYC	Royal Victoria Infirmary, Newcastle, UK	Clinical isolate (Throat- sputum)	Human infections	Admixed	Admixed
378604Xm	NCYC	Royal Victoria Infirmary, Newcastle, UK	Clinical isolate (Sputum)	Human infections	Admixed	Admixed
AN1f2s1	Bensasson	Agios Nikolaos, Greece	Rotting fig with fly larvae	Fruit or flower	Admixed	Admixed
AN1g2s1	Bensasson	Agios Nikolaos, Greece	Rotting fig with fly larvae	Fruit or flower	Admixed	Admixed
AN3e1s1	Bensasson	Agios Nikolaos, Greece	Fig	Fruit or flower	Admixed	Wine
ARN019As1	Wray*	N. Carolina, USA	Grape, *Vitis vinifera* Chardonnay	Fruit or flower	Wine	Wine
ARN020As1	Wray*	N. Carolina, USA	Grape, *V. vinifera* Cabernet	Fruit or flower	Admixed	Wine
ARN022As1	Wray*	N. Carolina, USA	Grape, *V. vinifera* Syrah	Fruit or flower	Wine	Wine
ARN056As1	Wray*	N. Carolina, USA	Grape, *V. vinifera* Reisling	Fruit or flower	Admixed	Admixed
ARN179As1	Wray*	N. Carolina, USA	Grape, *V. vinifera* Sangiovese	Fruit or flower	NCO	NCO

Table S1 – continued from previous page

Name	Strain Source[a]	Geographic Origin	Habitat Origin	Habitat Category[b]	*structure* lineage[c]	Locus-by-locus lineage[c]
ARN202Bs1	Wray*	N. Carolina, USA	Grape, *V. vinifera* Syrah	Fruit or flower	NCO	NCO
ARN231As1	Wray*	N. Carolina, USA	Grape, *V. vinifera* Carlos	Fruit or flower	Admixed	Admixed
ARN239As1	Wray*	N. Carolina, USA	Grape, *V. vinifera* Carlos	Fruit or flower	NCO	NCO
ARN244As1	Wray*	N. Carolina, USA	Grape, *V. vinifera* Carlos	Fruit or flower	Admixed	Admixed
ARN245As1	Wray*	N. Carolina, USA	Grape, *V. vinifera* Carlos	Fruit or flower	Admixed	Admixed
BC187m	NCYC	Napa Valley, California, USA	Barrel fermentation	Fermentations	Admixed	Admixed
DBVPG1106m	NCYC	Australia	Grapes	Fruit or flower	Wine[R]	Wine[R]
DBVPG1373m	NCYC	Netherlands	Soil	Soil and unknown	Admixed	Wine
DBVPG1788m	NCYC	Turku, Finland	Soil	Soil and unknown	Admixed	Wine
DBVPG1853m	NCYC	Ethiopia, East Africa	White Teff	Fermentations	Admixed	Wine
DBVPG6044m	NCYC	West Africa	Bili wine, from *Osbeckia grandiflora*	Fermentations	WA[R]	WA[R]
DBVPG6765m	NCYC	Unknown	Unknown	Soil and unknown	Admixed	Wine

Table S1 – continued from previous page

Name	Strain Source[a]	Geographic Origin	Habitat Origin	Habitat Category[b]	*structure* lineage[c]	Locus-by-locus lineage[c]
HUN9s1	Barrio*	Debrecen, Hungary	Oak forest soil	Oak or similar trees	Admixed	EUO
K11m	NCYC	Japan	Shochu sake, Awamori	Fermentations	Sake[R]	Sake[R]
L-1374m	NCYC	Cauquenes, Chile	Fermentation from must	Fermentations	Wine[R]	Wine[R]
L-1528m	NCYC	Cauquenes, Chile	Fermentation from must	Fermentations	Wine	Wine
NCYC110m	NCYC	West Africa	Ginger beer from *Z. officinale*	Fermentations	WA[R]	WA[R]
NCYC361m	NCYC	Ireland	Beer spoilage strain from wort	Fermentations	Admixed	Admixed
PYR4b1s1	Bensasson	Agios Nikolaos, Greece	Oak bark, *Quercus pubescens*	Oak or similar trees	EUO	EUO
RM11-1a	Broad Institute	California, USA	Fermenting grape must	Fermentations	Admixed	Wine
S288cRef	SGD	Merced, California, USA	Rotting fig, and multiple crosses	Fruit or flower	Admixed	Admixed
SDO1s1	Diezmann*	N. Carolina, USA	Tulip tree soil, *Liriodendron tulipifera*	Oak or similar trees	NCO[R]	NCO[R]
SDO2s1	Diezmann*	N. Carolina, USA	Oak soil, *Q. prinus*	Oak or similar trees	NCO	NCO
SDO3s1	Diezmann*	N. Carolina, USA	*Gaultheria* sp. soil	Oak or similar trees	NCO[R]	NCO[R]

Table S1 – continued from previous page

Name	Strain Source[a]	Geographic Origin	Habitat Origin	Habitat Category[b]	*structure* lineage[c]	Locus-by-locus lineage[c]
SDO4s1	Diezmann*	N. Carolina, USA	Oak soil, *Q. prinus*	Oak or similar trees	NCO	NCO
SDO6s1	Diezmann*	N. Carolina, USA	Maple soil, *Acer* sp.	Oak or similar trees	NCO	NCO
SDO7s1	Diezmann*	N. Carolina, USA	Oak soil, *Q. prinus*	Oak or similar trees	NCO	NCO
SDO8s1	Diezmann*	N. Carolina, USA	Oak soil, *Q. prinus*	Oak or similar trees	NCO	Admixed
SDO9s1	Diezmann*	N. Carolina, USA	Oak soil, *Q. prinus*	Oak or similar trees	NCO	Admixed
SK1m	NCYC	USA	Soil	Soil and unknown	Admixed	Admixed
SM01s1	Diezmann*	N. Carolina, USA	Oak soil, *Q. alba*	Oak or similar trees	Admixed	Admixed
SM02s1	Diezmann*	N. Carolina, USA	Oak soil, *Q. alba*	Oak or similar trees	Admixed	Admixed
SM12s1	Diezmann*	N. Carolina, USA	Maple soil, *Acer* sp.	Oak or similar trees	NCO	NCO
SM17s1	Diezmann*	N. Carolina, USA	Oak soil, *Q. alba*	Oak or similar trees	NCO	NCO
SM66s1	Diezmann*	N. Carolina, USA	Oak soil, *Q. alba*	Oak or similar trees	NCO	NCO
SM69s1	Diezmann*	N. Carolina, USA	Oak soil, *Q. prinus*	Oak or similar trees	NCO	NCO
UWOPS03-461.4m	NCYC	Telok Senangin, Malaysia	Nectar, Bertram palm	Fruit or flower	MAL[R]	MAL[R]

Table S1 – continued from previous page

Name	Strain Source[a]	Geographic Origin	Habitat Origin	Habitat Category[b]	*structure* lineage[c]	Locus-by-locus lineage[c]
UWOPS05-217.3m	NCYC	Telok Senangin, Malaysia	Nectar, Bertram palm	Fruit or flower	MAL[R]	MAL[R]
UWOPS05-227.2m	NCYC	Telok Senangin, Malaysia	Nectar, Bertram palm	Fruit or flower	MAL	MAL
UWOPS83-787.3m	NCYC	Great Inagua Island, Bahamas	Fruit, *Opuntia stricta*	Fruit or flower	Admixed	Admixed
UWOPS87-2421m	NCYC	Puhelu Road, Maui, Hawaii	Cladode, *Opuntia megacantha*	Fruit or flower	Admixed	Admixed
Y12m	NCYC	Ivory Coast, West Africa	Palm wine	Fermentations	Sake	Sake
Y55m	NCYC	France	Grape	Fruit or flower	Admixed	Admixed
Y9m	NCYC	Indonesia	Ragi	Fermentations	Sake[R]	Sake[R]
YIIc17_E5m	NCYC	Sauternes, France	Wine	Fermentations	Admixed	Admixed
YJM789	Wei et al 2007	Missouri, USA	lung of AIDS patient with pneumonia	Human infections	Admixed	Admixed
YJM975m	NCYC	Ospedali Riuniti di Bergamo, Italy	Vagina of patient suffering from vaginitis	Human infections	Wine	Wine
YJM978m	NCYC	Ospedali Riuniti di Bergamo, Italy	Vagina of patient suffering from vaginitis	Human infections	Wine	Wine

Table S1 – continued from previous page

Name	Strain Source[a]	Geographic Origin	Habitat Origin	Habitat Category[b]	*structure* lineage[c]	Locus-by-locus lineage[c]
YJM981m	NCYC	Ospedali Riuniti di Bergamo, Italy	Vagina of patient suffering from vaginitis	Human infections	Wine	Wine
YPS128m	NCYC	Pennsylvania, USA	Oak soil, *Q. alba*	Oak or similar trees	PAO[R]	PAO[R]
YPS139s1	Sniegowski*	Pennsylvania, USA	Oak soil, *Quercus* sp.	Oak or similar trees	PAO	PAO
YPS396	Sniegowski	Pennsylvania, USA	Oak soil, *Q. velutina*	Oak or similar trees	PAO	PAO
YPS400	Sniegowski	Pennsylvania, USA	Oak soil, *Q. rubra*	Oak or similar trees	PAO	PAO
YPS600	Sniegowski	Pennsylvania, USA	Oak flux, *Q. alba*	Oak or similar trees	PAO	PAO
YPS602	Sniegowski	Pennsylvania, USA	Oak soil, *Q. alba*	Oak or similar trees	PAO	PAO
YPS604	Sniegowski	Pennsylvania, USA	Oak soil, *Q. velutina*	Oak or similar trees	PAO	PAO
YPS606m	NCYC	Pennsylvania, USA	Oak bark, *Q. rubra*	Oak or similar trees	PAO[R]	PAO[R]
YPS608	Sniegowski	Pennsylvania, USA	Oak soil, *Q. rubra*	Oak or similar trees	PAO	PAO
YPS610	Sniegowski	Pennsylvania, USA	Oak bark, *Q. velutina*	Oak or similar trees	PAO	PAO
ZP560s1	Sampaio*	Castelo de Vide, Portugal	Oak bark, *Q. pyrenaica*	Oak or similar trees	EUO[R]	EUO[R]

Table S1 – continued from previous page

Name	Strain Source[a]	Geographic Origin	Habitat Origin	Habitat Category[b]	*structure* lineage[c]	Locus-by-locus lineage[c]
ZP561s1	Sampaio*	Castelo de Vide, Portugal	Oak bark, *Q. pyrenaica*	Oak or similar trees	EUO	EUO
ZP562s1	Sampaio*	Castelo de Vide, Portugal	Oak bark, *Q. ilex*	Oak or similar trees	EUO[R]	EUO[R]
ZP565s1	Sampaio*	Castelo de Vide, Portugal	Chestnut bark, *Castanea sativa*	Oak or similar trees	EUO	EUO
ZP568s1	Sampaio*	Castelo de Vide, Portugal	Oak bark, *Q. pyrenaica*	Oak or similar trees	EUO	EUO
ZP577s1	Sampaio*	Aldeia das Dez, Portugal	Oak bark, *Q. faginea*	Oak or similar trees	Wine	Wine
ZP578s1	Sampaio*	Aldeia das Dez, Portugal	Oak bark, *Q. faginea*	Oak or similar trees	Wine	Wine
ZP579s1	Sampaio*	Aldeia das Dez, Portugal	Oak bark, *Q. pyrenaica*	Oak or similar trees	Admixed	Wine
ZP633s1	Sampaio*	Castelo de Vide, Portugal	Oak bark, *Q. pyrenaica*	Oak or similar trees	EUO	EUO
ZP636s1	Sampaio*	Castelo de Vide, Portugal	Chestnut bark, *Castanea sativa*	Oak or similar trees	EUO	EUO

* We generated monosporic derivative strains from the original strain.

[a] The source of the parental strain.

[b] The categories used in Table 2.

[c] Lineage determined by *structure* or locus-by-locus phylogenetic analysis: NCO is North Carolina Oak; PAO is Pennsylvanian Oak; EUO is European Oak; MAL is Malaysian; and WA is West African.

[R] This strain was used as a reference strain for this lineage.
