# Supplementary material for: Habitat Predicts Levels of Genetic Admixture in Saccharomyces cerevisiae
Source: G3 (Bethesda). 2017 Jul 10;7(9):2919–29. doi: 10.1534/g3.117.041806 (PMC5592920; doi:10.1534/g3.117.041806)
Supplement: Supplementary file 3 [file 2919FileS3.pdf]

File S3: **Locus by locus trees in .pdf format showing the phylogenetic trees obtained for all 16 centromeres**. Centromere loci are labelled CEN1 to CEN16, strains used as reference strains for their population are labelled with a coloured dot and strain names are coloured according to the final population assignment based on the locus by locus analysis of these trees: admixed strains are shown in black, blue is Malaysia, purple is European Wine, orange is West African, red is Sake, dark green with “YPS” prefix is Pennsylvania oak, mid-green with “SD”, “SM” or “ARN” prefix is North Carolina Oak, light green with a “ZP” prefix is European Oak. Trees were constructed from F84 distances using the neighbour joining method, and bootstrap values are percentages based on 10,000 bootstrap replicates. The phylogenies for every locus show that DBVPG1853m is most similar to European strains (10 out of 16 centromeres). At three loci (CEN3, 5 and 7) there is also bootstrap support (at least 80%) that this strain represents the closest outgroup to the Wine and European Oak populations.

CEN1

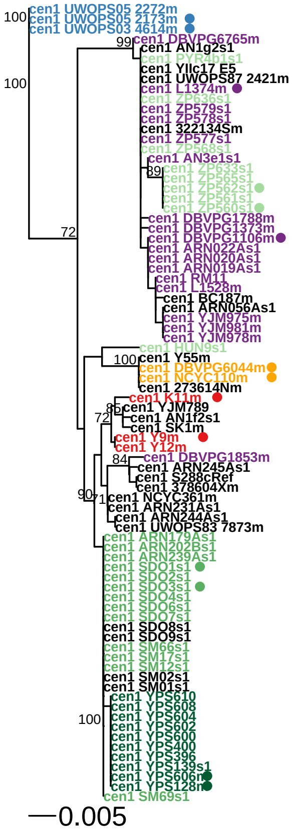

CEN2

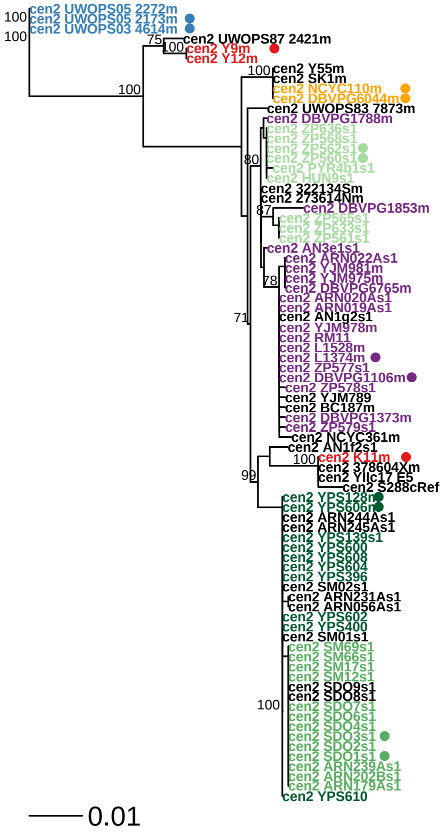

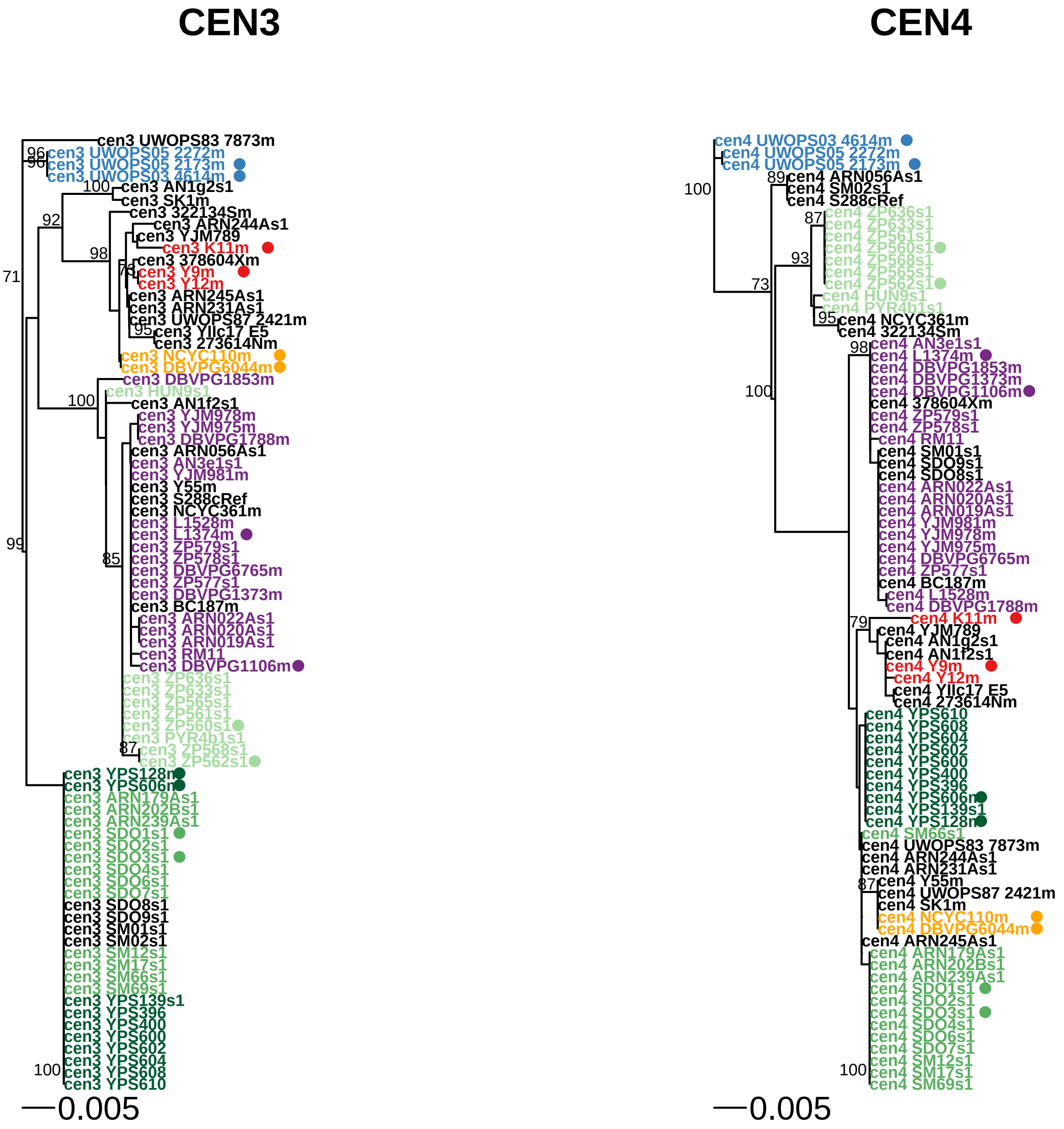

CEN3
cen3 UWOPS83 7873m
96
cen3 UWOPS05 2272m
96
cen3 UWOPS05 2173m
cen3 UWOPS03 4614m
100
cen3 AN1g2s1
cen3 SK1m
92
cen3 322134Sm
cen3 ARN244As1
cen3 YJM789
98
cen3 K11m
cen3 378604Xm
71
78
cen3 Y9m
cen3 Y12m
cen3 ARN245As1
cen3 ARN231As1
cen3 UWOPS87 2421m
95
cen3 YIIc17 E5
cen3 273614Nm
cen3 NCYC110m
cen3 DBVPG6044m
cen3 DBVPG1853m
cen3 HUN9s1
100
cen3 AN1f2s1
cen3 YJM978m
cen3 YJM975m
cen3 DBVPG1788m
cen3 ARN056As1
cen3 AN3e1s1
cen3 YJM981m
cen3 Y55m
cen3 S288cRef
cen3 NCYC361m
cen3 L1528m
cen3 L1374m
99
cen3 ZP579s1
85
cen3 ZP578s1
cen3 DBVPG6765m
cen3 ZP577s1
cen3 DBVPG1373m
cen3 BC187m
cen3 ARN022As1
cen3 ARN020As1
cen3 ARN019As1
cen3 RM11
cen3 DBVPG1106m
cen3 ZP636s1
cen3 ZP633s1
cen3 ZP565s1
cen3 ZP561s1
cen3 ZP560s1
cen3 PYR4b1s1
87
cen3 ZP568s1
cen3 ZP562s1
cen3 YPS128m
cen3 YPS606m
cen3 ARN179As1
cen3 ARN202Bs1
cen3 ARN239As1
cen3 SDO1s1
cen3 SDO2s1
cen3 SDO3s1
cen3 SDO4s1
cen3 SDO6s1
cen3 SDO7s1
cen3 SDO8s1
cen3 SDO9s1
cen3 SM01s1
cen3 SM02s1
cen3 SM12s1
cen3 SM17s1
cen3 SM66s1
cen3 SM69s1
cen3 YPS139s1
cen3 YPS396
cen3 YPS400
cen3 YPS600
cen3 YPS602
cen3 YPS604
100
cen3 YPS608
cen3 YPS610
0.005
CEN4
cen4 UWOPS03 4614m
cen4 UWOPS05 2272m
cen4 UWOPS05 2173m
89
cen4 ARN056As1
100
cen4 SM02s1
cen4 S288cRef
87
cen4 ZP636s1
cen4 ZP633s1
cen4 ZP561s1
cen4 ZP560s1
93
cen4 ZP568s1
cen4 ZP565s1
73
cen4 ZP562s1
cen4 HUN9s1
cen4 PYR4b1s1
95
cen4 NCYC361m
cen4 322134Sm
98
cen4 AN3e1s1
cen4 L1374m
cen4 DBVPG1853m
cen4 DBVPG1373m
100
cen4 DBVPG1106m
cen4 378604Xm
cen4 ZP579s1
cen4 ZP578s1
cen4 RM11
cen4 SM01s1
cen4 SDO9s1
cen4 SDO8s1
cen4 ARN022As1
cen4 ARN020As1
cen4 ARN019As1
cen4 YJM981m
cen4 YJM978m
cen4 YJM975m
cen4 DBVPG6765m
cen4 ZP577s1
cen4 BC187m
cen4 L1528m
cen4 DBVPG1788m
79
cen4 K11m
cen4 YJM789
cen4 AN1g2s1
cen4 AN1f2s1
cen4 Y9m
cen4 Y12m
cen4 YIIc17 E5
cen4 273614Nm
cen4 YPS610
cen4 YPS608
cen4 YPS604
cen4 YPS602
cen4 YPS600
cen4 YPS400
cen4 YPS396
cen4 YPS606m
cen4 YPS139s1
cen4 YPS128m
cen4 SM66s1
cen4 UWOPS83 7873m
cen4 ARN244As1
cen4 ARN231As1
87
cen4 Y55m
cen4 UWOPS87 2421m
cen4 SK1m
cen4 NCYC110m
cen4 DBVPG6044m
cen4 ARN245As1
cen4 ARN179As1
cen4 ARN202Bs1
cen4 ARN239As1
cen4 SDO1s1
cen4 SDO2s1
cen4 SDO3s1
cen4 SDO4s1
cen4 SDO6s1
cen4 SDO7s1
cen4 SM12s1
100
cen4 SM17s1
cen4 SM69s1
0.005

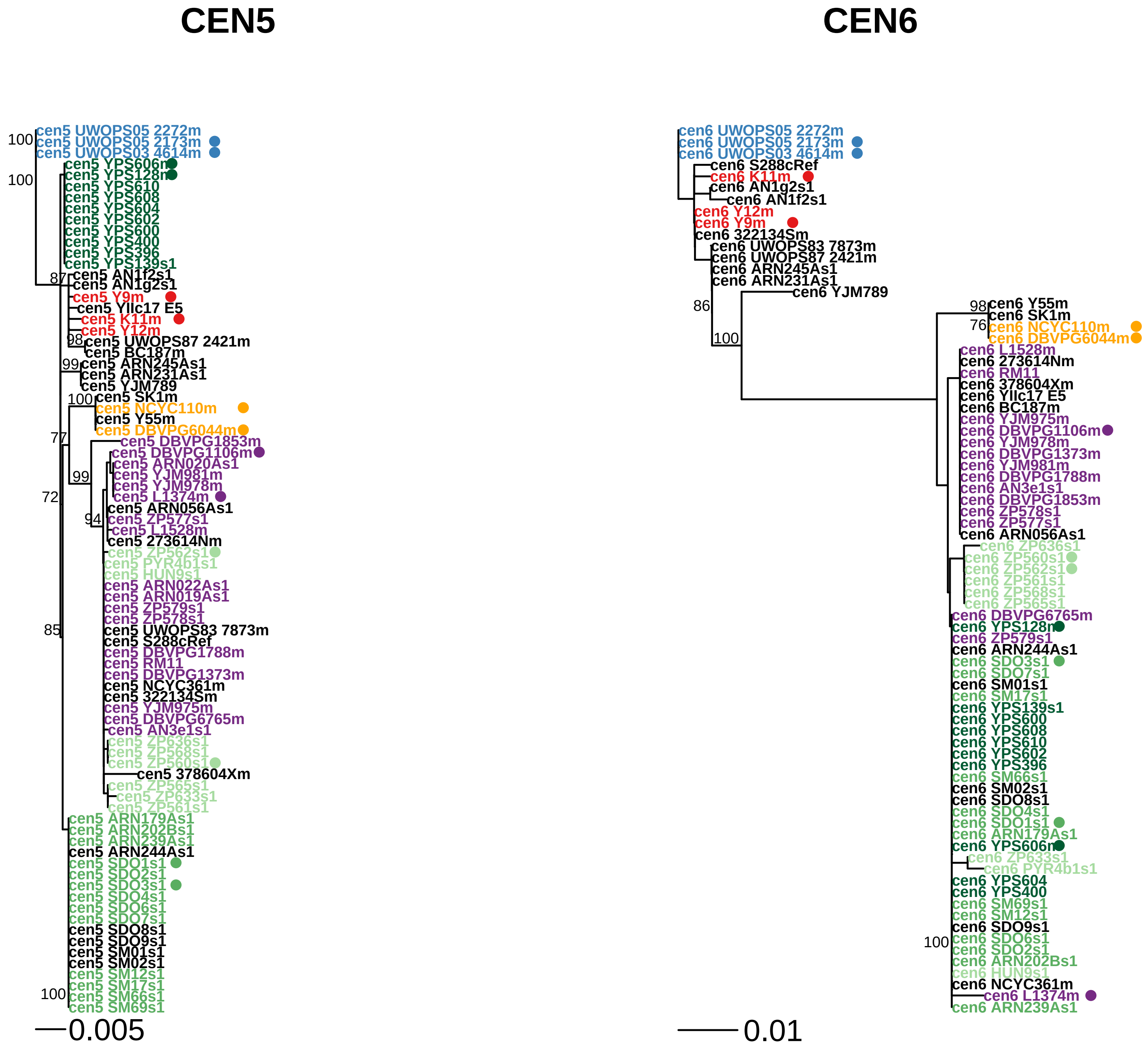

CEN5
cen5 UWOPS05 2272m
cen5 UWOPS05 2173m
cen5 UWOPS03 4614m
cen5 YPS606m
cen5 YPS128m
cen5 YPS610
cen5 YPS608
cen5 YPS604
cen5 YPS602
cen5 YPS600
cen5 YPS400
cen5 YPS396
cen5 YPS139s1
cen5 AN1f2s1
cen5 AN1g2s1
cen5 Y9m
cen5 YIIc17 E5
cen5 K11m
cen5 Y12m
cen5 UWOPS87 2421m
cen5 BC187m
cen5 ARN245As1
cen5 ARN231As1
cen5 YJM789
cen5 SK1m
cen5 NCYC110m
cen5 Y55m
cen5 DBVPG6044m
cen5 DBVPG1853m
cen5 DBVPG1106m
cen5 ARN020As1
cen5 YJM981m
cen5 YJM978m
cen5 L1374m
cen5 ARN056As1
cen5 ZP577s1
cen5 L1528m
cen5 273614Nm
cen5 ZP562s1
cen5 PYR4b1s1
cen5 HUN9s1
cen5 ARN022As1
cen5 ARN019As1
cen5 ZP579s1
cen5 ZP578s1
cen5 UWOPS83 7873m
cen5 S288cRef
cen5 DBVPG1788m
cen5 RM11
cen5 DBVPG1373m
cen5 NCYC361m
cen5 322134Sm
cen5 YJM975m
cen5 DBVPG6765m
cen5 AN3e1s1
cen5 ZP636s1
cen5 ZP568s1
cen5 ZP560s1
cen5 378604Xm
cen5 ZP565s1
cen5 ZP633s1
cen5 ZP561s1
cen5 ARN179As1
cen5 ARN202Bs1
cen5 ARN239As1
cen5 ARN244As1
cen5 SDO1s1
cen5 SDO2s1
cen5 SDO3s1
cen5 SDO4s1
cen5 SDO6s1
cen5 SDO7s1
cen5 SDO8s1
cen5 SDO9s1
cen5 SM01s1
cen5 SM02s1
cen5 SM12s1
cen5 SM17s1
cen5 SM66s1
cen5 SM69s1
100
100
87
98
99
100
77
99
72
94
85
100
0.005
CEN6
cen6 UWOPS05 2272m
cen6 UWOPS05 2173m
cen6 UWOPS03 4614m
cen6 S288cRef
cen6 K11m
cen6 AN1g2s1
cen6 AN1f2s1
cen6 Y12m
cen6 Y9m
cen6 322134Sm
cen6 UWOPS83 7873m
cen6 UWOPS87 2421m
cen6 ARN245As1
cen6 ARN231As1
cen6 YJM789
cen6 Y55m
cen6 SK1m
cen6 NCYC110m
cen6 DBVPG6044m
cen6 L1528m
cen6 273614Nm
cen6 RM11
cen6 378604Xm
cen6 YIIc17 E5
cen6 BC187m
cen6 YJM975m
cen6 DBVPG1106m
cen6 YJM978m
cen6 DBVPG1373m
cen6 YJM981m
cen6 DBVPG1788m
cen6 AN3e1s1
cen6 DBVPG1853m
cen6 ZP578s1
cen6 ZP577s1
cen6 ARN056As1
cen6 ZP636s1
cen6 ZP560s1
cen6 ZP562s1
cen6 ZP561s1
cen6 ZP568s1
cen6 ZP565s1
cen6 DBVPG6765m
cen6 YPS128m
cen6 ZP579s1
cen6 ARN244As1
cen6 SDO3s1
cen6 SDO7s1
cen6 SM01s1
cen6 SM17s1
cen6 YPS139s1
cen6 YPS600
cen6 YPS608
cen6 YPS610
cen6 YPS602
cen6 YPS396
cen6 SM66s1
cen6 SM02s1
cen6 SDO8s1
cen6 SDO4s1
cen6 SDO1s1
cen6 ARN179As1
cen6 YPS606m
cen6 ZP633s1
cen6 PYR4b1s1
cen6 YPS604
cen6 YPS400
cen6 SM69s1
cen6 SM12s1
cen6 SDO9s1
cen6 SDO6s1
cen6 SDO2s1
cen6 ARN202Bs1
cen6 HUN9s1
cen6 NCYC361m
cen6 L1374m
cen6 ARN239As1
86
100
98
76
100
0.01

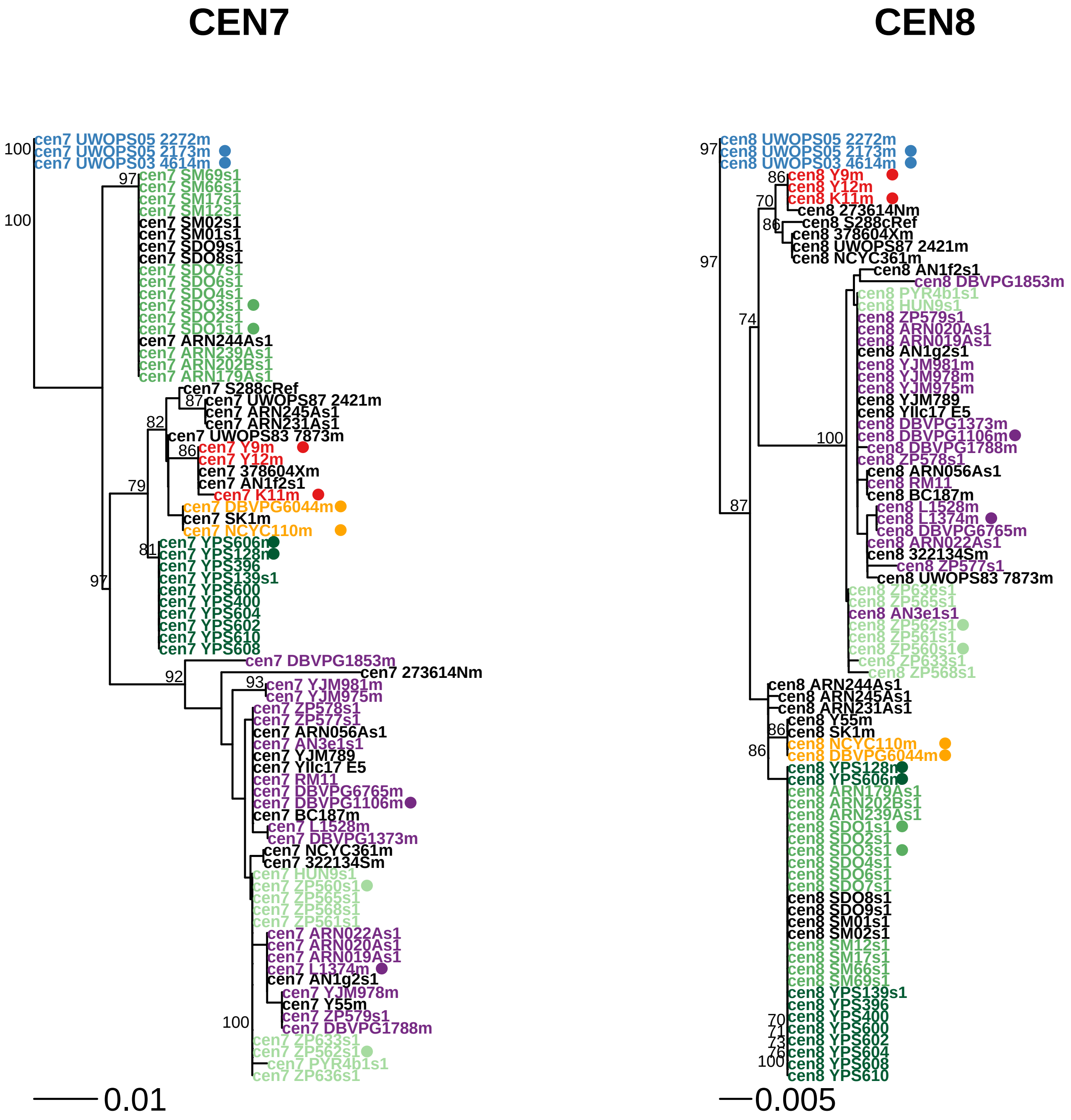
CEN7
cen7 UWOPS05 2272m
cen7 UWOPS05 2173m
cen7 UWOPS03 4614m
cen7 SM69s1
cen7 SM66s1
cen7 SM17s1
cen7 SM12s1
cen7 SM02s1
cen7 SM01s1
cen7 SDO9s1
cen7 SDO8s1
cen7 SDO7s1
cen7 SDO6s1
cen7 SDO4s1
cen7 SDO3s1
cen7 SDO2s1
cen7 SDO1s1
cen7 ARN244As1
cen7 ARN239As1
cen7 ARN202Bs1
cen7 ARN179As1
cen7 S288cRef
cen7 UWOPS87 2421m
cen7 ARN245As1
cen7 ARN231As1
cen7 UWOPS83 7873m
cen7 Y9m
cen7 Y12m
cen7 378604Xm
cen7 AN1f2s1
cen7 K11m
cen7 DBVPG6044m
cen7 SK1m
cen7 NCYC110m
cen7 YPS606m
cen7 YPS128m
cen7 YPS396
cen7 YPS139s1
cen7 YPS600
cen7 YPS400
cen7 YPS604
cen7 YPS602
cen7 YPS610
cen7 YPS608
cen7 DBVPG1853m
cen7 273614Nm
cen7 YJM981m
cen7 YJM975m
cen7 ZP578s1
cen7 ZP577s1
cen7 ARN056As1
cen7 AN3e1s1
cen7 YJM789
cen7 YIIc17 E5
cen7 RM11
cen7 DBVPG6765m
cen7 DBVPG1106m
cen7 BC187m
cen7 L1528m
cen7 DBVPG1373m
cen7 NCYC361m
cen7 322134Sm
cen7 HUN9s1
cen7 ZP560s1
cen7 ZP565s1
cen7 ZP568s1
cen7 ZP561s1
cen7 ARN022As1
cen7 ARN020As1
cen7 ARN019As1
cen7 L1374m
cen7 AN1g2s1
cen7 YJM978m
cen7 Y55m
cen7 ZP579s1
cen7 DBVPG1788m
cen7 ZP633s1
cen7 ZP562s1
cen7 PYR4b1s1
cen7 ZP636s1
100
100
97
97
82
87
86
79
81
92
93
100
0.01
CEN8
cen8 UWOPS05 2272m
cen8 UWOPS05 2173m
cen8 UWOPS03 4614m
cen8 Y9m
cen8 Y12m
cen8 K11m
cen8 273614Nm
cen8 S288cRef
cen8 378604Xm
cen8 UWOPS87 2421m
cen8 NCYC361m
cen8 AN1f2s1
cen8 DBVPG1853m
cen8 PYR4b1s1
cen8 HUN9s1
cen8 ZP579s1
cen8 ARN020As1
cen8 ARN019As1
cen8 AN1g2s1
cen8 YJM981m
cen8 YJM978m
cen8 YJM975m
cen8 YJM789
cen8 YIIc17 E5
cen8 DBVPG1373m
cen8 DBVPG1106m
cen8 DBVPG1788m
cen8 ZP578s1
cen8 ARN056As1
cen8 RM11
cen8 BC187m
cen8 L1528m
cen8 L1374m
cen8 DBVPG6765m
cen8 ARN022As1
cen8 322134Sm
cen8 ZP577s1
cen8 UWOPS83 7873m
cen8 ZP636s1
cen8 ZP565s1
cen8 AN3e1s1
cen8 ZP562s1
cen8 ZP561s1
cen8 ZP560s1
cen8 ZP633s1
cen8 ZP568s1
cen8 ARN244As1
cen8 ARN245As1
cen8 ARN231As1
cen8 Y55m
cen8 SK1m
cen8 NCYC110m
cen8 DBVPG6044m
cen8 YPS128m
cen8 YPS606m
cen8 ARN179As1
cen8 ARN202Bs1
cen8 ARN239As1
cen8 SDO1s1
cen8 SDO2s1
cen8 SDO3s1
cen8 SDO4s1
cen8 SDO6s1
cen8 SDO7s1
cen8 SDO8s1
cen8 SDO9s1
cen8 SM01s1
cen8 SM02s1
cen8 SM12s1
cen8 SM17s1
cen8 SM66s1
cen8 SM69s1
cen8 YPS139s1
cen8 YPS396
cen8 YPS400
cen8 YPS600
cen8 YPS602
cen8 YPS604
cen8 YPS608
cen8 YPS610
97
86
70
86
97
74
100
87
86
86
70
71
73
76
100
0.005

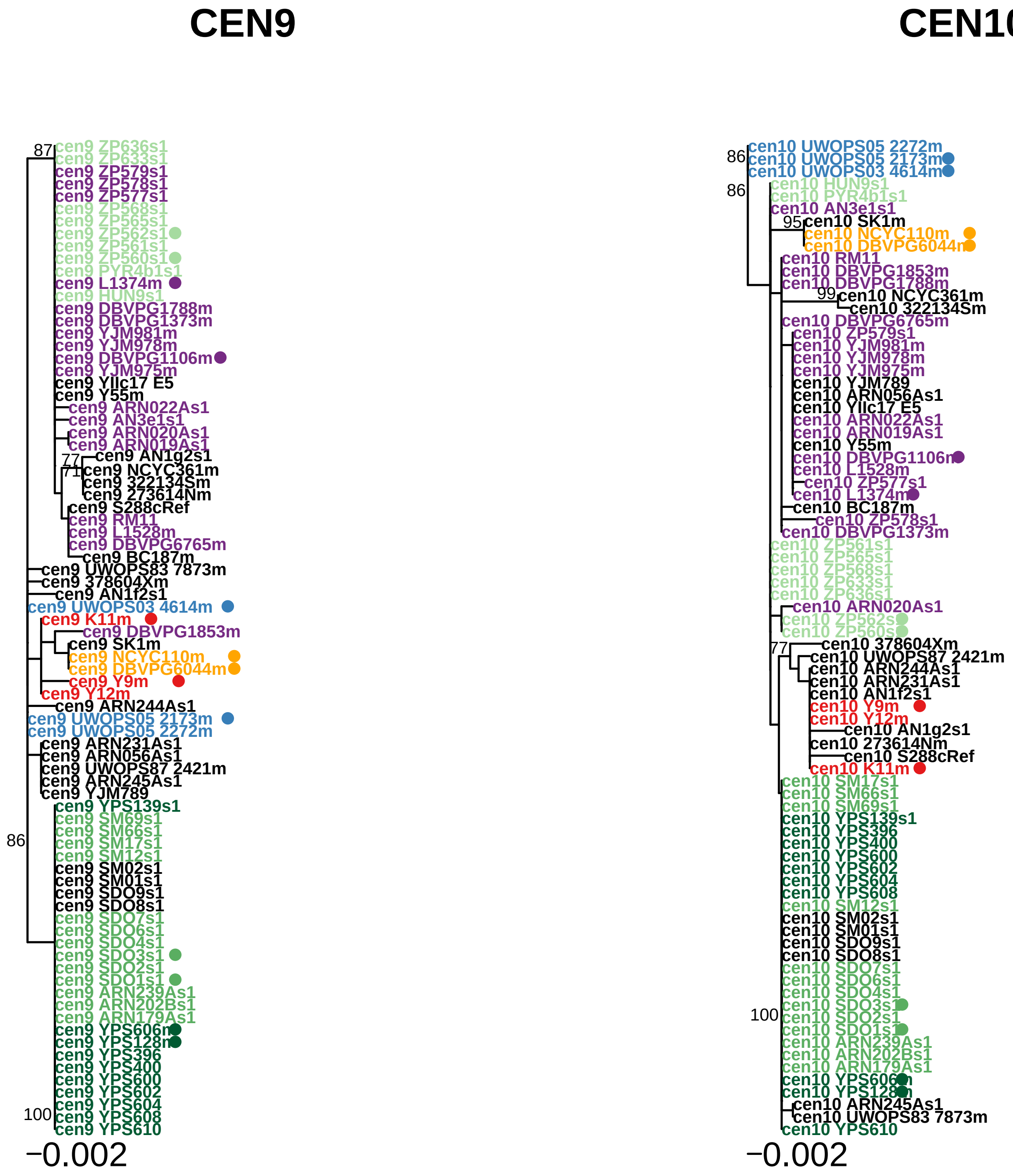
CEN9
87
cen9 ZP636s1
cen9 ZP633s1
cen9 ZP579s1
cen9 ZP578s1
cen9 ZP577s1
cen9 ZP568s1
cen9 ZP565s1
cen9 ZP562s1
cen9 ZP561s1
cen9 ZP560s1
cen9 PYR4b1s1
cen9 L1374m
cen9 HUN9s1
cen9 DBVPG1788m
cen9 DBVPG1373m
cen9 YJM981m
cen9 YJM978m
cen9 DBVPG1106m
cen9 YJM975m
cen9 YIIc17 E5
cen9 Y55m
cen9 ARN022As1
cen9 AN3e1s1
cen9 ARN020As1
cen9 ARN019As1
77
cen9 AN1g2s1
71
cen9 NCYC361m
cen9 322134Sm
cen9 273614Nm
cen9 S288cRef
cen9 RM11
cen9 L1528m
cen9 DBVPG6765m
cen9 BC187m
cen9 UWOPS83 7873m
cen9 378604Xm
cen9 AN1f2s1
cen9 UWOPS03 4614m
cen9 K11m
cen9 DBVPG1853m
cen9 SK1m
cen9 NCYC110m
cen9 DBVPG6044m
cen9 Y9m
cen9 Y12m
cen9 ARN244As1
cen9 UWOPS05 2173m
cen9 UWOPS05 2272m
cen9 ARN231As1
cen9 ARN056As1
cen9 UWOPS87 2421m
cen9 ARN245As1
cen9 YJM789
cen9 YPS139s1
86
cen9 SM69s1
cen9 SM66s1
cen9 SM17s1
cen9 SM12s1
cen9 SM02s1
cen9 SM01s1
cen9 SDO9s1
cen9 SDO8s1
cen9 SDO7s1
cen9 SDO6s1
cen9 SDO4s1
cen9 SDO3s1
cen9 SDO2s1
cen9 SDO1s1
cen9 ARN239As1
cen9 ARN202Bs1
cen9 ARN179As1
cen9 YPS606m
cen9 YPS128m
cen9 YPS396
cen9 YPS400
cen9 YPS600
cen9 YPS602
cen9 YPS604
100
cen9 YPS608
cen9 YPS610
−0.002
CEN10
cen10 UWOPS05 2272m
86
cen10 UWOPS05 2173m
cen10 UWOPS03 4614m
86
cen10 HUN9s1
cen10 PYR4b1s1
cen10 AN3e1s1
95
cen10 SK1m
cen10 NCYC110m
cen10 DBVPG6044m
cen10 RM11
cen10 DBVPG1853m
cen10 DBVPG1788m
99
cen10 NCYC361m
cen10 322134Sm
cen10 DBVPG6765m
cen10 ZP579s1
cen10 YJM981m
cen10 YJM978m
cen10 YJM975m
cen10 YJM789
cen10 ARN056As1
cen10 YIIc17 E5
cen10 ARN022As1
cen10 ARN019As1
cen10 Y55m
cen10 DBVPG1106m
cen10 L1528m
cen10 ZP577s1
cen10 L1374m
cen10 BC187m
cen10 ZP578s1
cen10 DBVPG1373m
cen10 ZP561s1
cen10 ZP565s1
cen10 ZP568s1
cen10 ZP633s1
cen10 ZP636s1
cen10 ARN020As1
cen10 ZP562s1
cen10 ZP560s1
77
cen10 378604Xm
cen10 UWOPS87 2421m
cen10 ARN244As1
cen10 ARN231As1
cen10 AN1f2s1
cen10 Y9m
cen10 Y12m
cen10 AN1g2s1
cen10 273614Nm
cen10 S288cRef
cen10 K11m
cen10 SM17s1
cen10 SM66s1
cen10 SM69s1
cen10 YPS139s1
cen10 YPS396
cen10 YPS400
cen10 YPS600
cen10 YPS602
cen10 YPS604
cen10 YPS608
cen10 SM12s1
cen10 SM02s1
cen10 SM01s1
cen10 SDO9s1
cen10 SDO8s1
cen10 SDO7s1
cen10 SDO6s1
cen10 SDO4s1
100
cen10 SDO3s1
cen10 SDO2s1
cen10 SDO1s1
cen10 ARN239As1
cen10 ARN202Bs1
cen10 ARN179As1
cen10 YPS606m
cen10 YPS128m
cen10 ARN245As1
cen10 UWOPS83 7873m
cen10 YPS610
−0.002

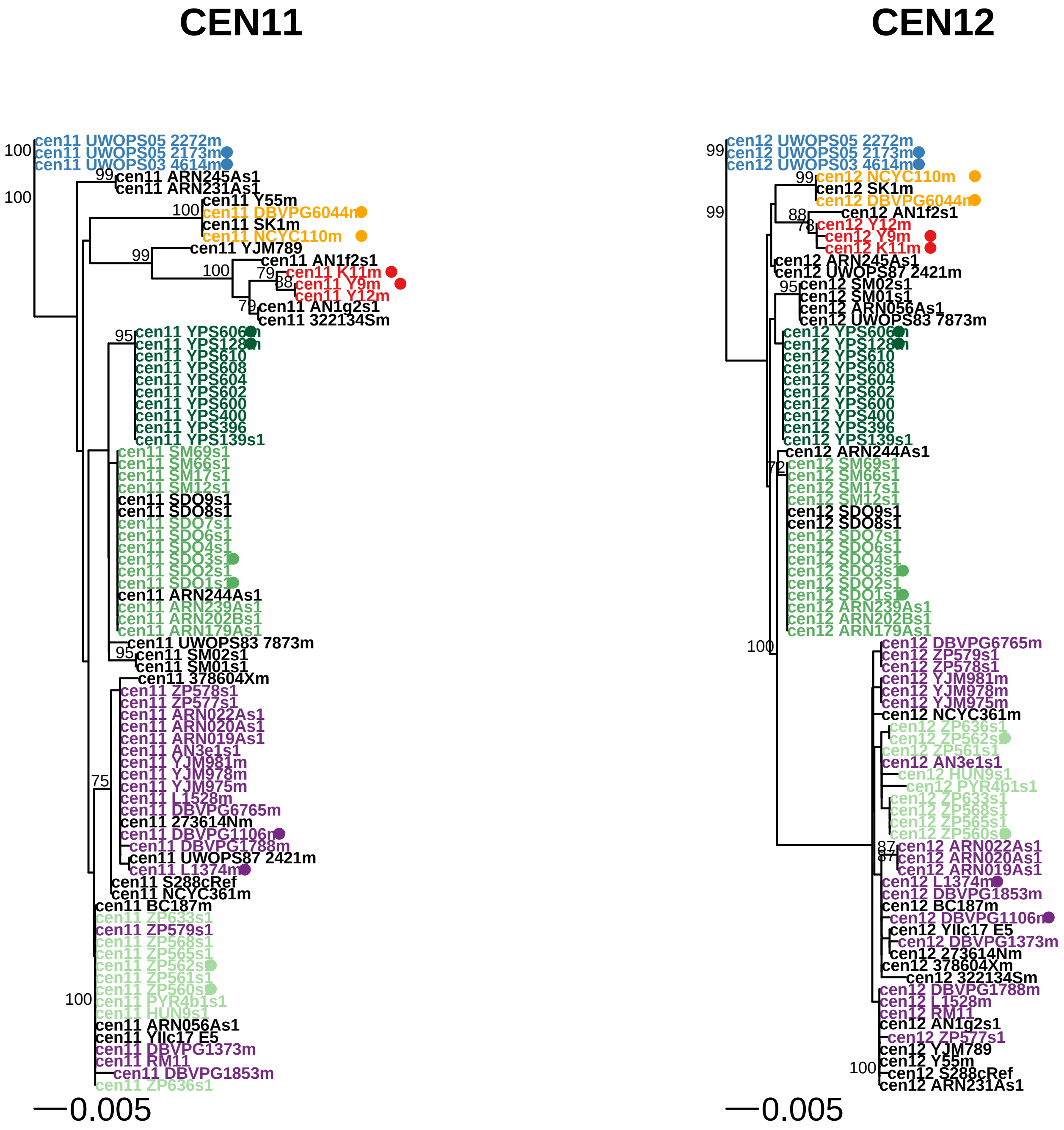
CEN11
CEN12
cen11 UWOPS05 2272m
cen11 UWOPS05 2173m
cen11 UWOPS03 4614m
cen11 ARN245As1
cen11 ARN231As1
cen11 Y55m
cen11 DBVPG6044m
cen11 SK1m
cen11 NCYC110m
cen11 YJM789
cen11 AN1f2s1
cen11 K11m
cen11 Y9m
cen11 Y12m
cen11 AN1g2s1
cen11 322134Sm
cen11 YPS606m
cen11 YPS128m
cen11 YPS610
cen11 YPS608
cen11 YPS604
cen11 YPS602
cen11 YPS600
cen11 YPS400
cen11 YPS396
cen11 YPS139s1
cen11 SM69s1
cen11 SM66s1
cen11 SM17s1
cen11 SM12s1
cen11 SDO9s1
cen11 SDO8s1
cen11 SDO7s1
cen11 SDO6s1
cen11 SDO4s1
cen11 SDO3s1
cen11 SDO2s1
cen11 SDO1s1
cen11 ARN244As1
cen11 ARN239As1
cen11 ARN202Bs1
cen11 ARN179As1
cen11 UWOPS83 7873m
cen11 SM02s1
cen11 SM01s1
cen11 378604Xm
cen11 ZP578s1
cen11 ZP577s1
cen11 ARN022As1
cen11 ARN020As1
cen11 ARN019As1
cen11 AN3e1s1
cen11 YJM981m
cen11 YJM978m
cen11 YJM975m
cen11 L1528m
cen11 DBVPG6765m
cen11 273614Nm
cen11 DBVPG1106m
cen11 DBVPG1788m
cen11 UWOPS87 2421m
cen11 L1374m
cen11 S288cRef
cen11 NCYC361m
cen11 BC187m
cen11 ZP633s1
cen11 ZP579s1
cen11 ZP568s1
cen11 ZP565s1
cen11 ZP562s1
cen11 ZP561s1
cen11 ZP560s1
cen11 PYR4b1s1
cen11 HUN9s1
cen11 ARN056As1
cen11 YIIc17 E5
cen11 DBVPG1373m
cen11 RM11
cen11 DBVPG1853m
cen11 ZP636s1
0.005
cen12 UWOPS05 2272m
cen12 UWOPS05 2173m
cen12 UWOPS03 4614m
cen12 NCYC110m
cen12 SK1m
cen12 DBVPG6044m
cen12 AN1f2s1
cen12 Y12m
cen12 Y9m
cen12 K11m
cen12 ARN245As1
cen12 UWOPS87 2421m
cen12 SM02s1
cen12 SM01s1
cen12 ARN056As1
cen12 UWOPS83 7873m
cen12 YPS606m
cen12 YPS128m
cen12 YPS610
cen12 YPS608
cen12 YPS604
cen12 YPS602
cen12 YPS600
cen12 YPS400
cen12 YPS396
cen12 YPS139s1
cen12 ARN244As1
cen12 SM69s1
cen12 SM66s1
cen12 SM17s1
cen12 SM12s1
cen12 SDO9s1
cen12 SDO8s1
cen12 SDO7s1
cen12 SDO6s1
cen12 SDO4s1
cen12 SDO3s1
cen12 SDO2s1
cen12 SDO1s1
cen12 ARN239As1
cen12 ARN202Bs1
cen12 ARN179As1
cen12 DBVPG6765m
cen12 ZP579s1
cen12 ZP578s1
cen12 YJM981m
cen12 YJM978m
cen12 YJM975m
cen12 NCYC361m
cen12 ZP636s1
cen12 ZP562s1
cen12 ZP561s1
cen12 AN3e1s1
cen12 HUN9s1
cen12 PYR4b1s1
cen12 ZP633s1
cen12 ZP568s1
cen12 ZP565s1
cen12 ZP560s1
cen12 ARN022As1
cen12 ARN020As1
cen12 ARN019As1
cen12 L1374m
cen12 DBVPG1853m
cen12 BC187m
cen12 DBVPG1106m
cen12 YIIc17 E5
cen12 DBVPG1373m
cen12 273614Nm
cen12 378604Xm
cen12 322134Sm
cen12 DBVPG1788m
cen12 L1528m
cen12 RM11
cen12 AN1g2s1
cen12 ZP577s1
cen12 YJM789
cen12 Y55m
cen12 S288cRef
cen12 ARN231As1
0.005

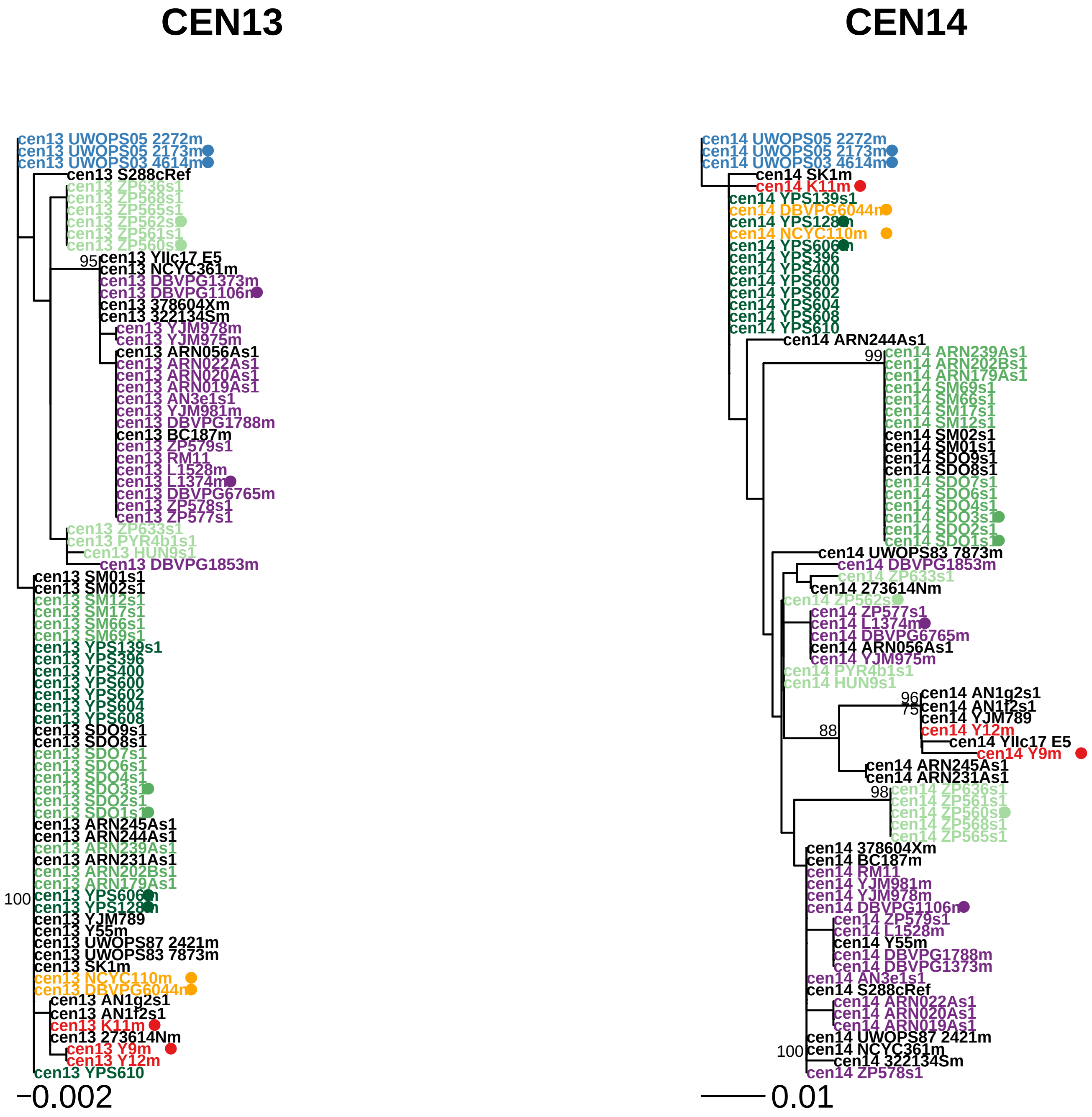
CEN13
CEN14
cen13 UWOPS05 2272m
cen13 UWOPS05 2173m
cen13 UWOPS03 4614m
cen13 S288cRef
cen13 ZP636s1
cen13 ZP568s1
cen13 ZP565s1
cen13 ZP562s1
cen13 ZP561s1
cen13 ZP560s1
95
cen13 YIIc17 E5
cen13 NCYC361m
cen13 DBVPG1373m
cen13 DBVPG1106m
cen13 378604Xm
cen13 322134Sm
cen13 YJM978m
cen13 YJM975m
cen13 ARN056As1
cen13 ARN022As1
cen13 ARN020As1
cen13 ARN019As1
cen13 AN3e1s1
cen13 YJM981m
cen13 DBVPG1788m
cen13 BC187m
cen13 ZP579s1
cen13 RM11
cen13 L1528m
cen13 L1374m
cen13 DBVPG6765m
cen13 ZP578s1
cen13 ZP577s1
cen13 ZP633s1
cen13 PYR4b1s1
cen13 HUN9s1
cen13 DBVPG1853m
cen13 SM01s1
cen13 SM02s1
cen13 SM12s1
cen13 SM17s1
cen13 SM66s1
cen13 SM69s1
cen13 YPS139s1
cen13 YPS396
cen13 YPS400
cen13 YPS600
cen13 YPS602
cen13 YPS604
cen13 YPS608
cen13 SDO9s1
cen13 SDO8s1
cen13 SDO7s1
cen13 SDO6s1
cen13 SDO4s1
cen13 SDO3s1
cen13 SDO2s1
cen13 SDO1s1
cen13 ARN245As1
cen13 ARN244As1
cen13 ARN239As1
cen13 ARN231As1
cen13 ARN202Bs1
cen13 ARN179As1
100
cen13 YPS606m
cen13 YPS128m
cen13 YJM789
cen13 Y55m
cen13 UWOPS87 2421m
cen13 UWOPS83 7873m
cen13 SK1m
cen13 NCYC110m
cen13 DBVPG6044m
cen13 AN1g2s1
cen13 AN1f2s1
cen13 K11m
cen13 273614Nm
cen13 Y9m
cen13 Y12m
cen13 YPS610
0.002
cen14 UWOPS05 2272m
cen14 UWOPS05 2173m
cen14 UWOPS03 4614m
cen14 SK1m
cen14 K11m
cen14 YPS139s1
cen14 DBVPG6044m
cen14 YPS128m
cen14 NCYC110m
cen14 YPS606m
cen14 YPS396
cen14 YPS400
cen14 YPS600
cen14 YPS602
cen14 YPS604
cen14 YPS608
cen14 YPS610
cen14 ARN244As1
99
cen14 ARN239As1
cen14 ARN202Bs1
cen14 ARN179As1
cen14 SM69s1
cen14 SM66s1
cen14 SM17s1
cen14 SM12s1
cen14 SM02s1
cen14 SM01s1
cen14 SDO9s1
cen14 SDO8s1
cen14 SDO7s1
cen14 SDO6s1
cen14 SDO4s1
cen14 SDO3s1
cen14 SDO2s1
cen14 SDO1s1
cen14 UWOPS83 7873m
cen14 DBVPG1853m
cen14 ZP633s1
cen14 273614Nm
cen14 ZP562s1
cen14 ZP577s1
cen14 L1374m
cen14 DBVPG6765m
cen14 ARN056As1
cen14 YJM975m
cen14 PYR4b1s1
cen14 HUN9s1
96
cen14 AN1g2s1
75
cen14 AN1f2s1
cen14 YJM789
88
cen14 Y12m
cen14 YIIc17 E5
cen14 Y9m
cen14 ARN245As1
cen14 ARN231As1
98
cen14 ZP636s1
cen14 ZP561s1
cen14 ZP560s1
cen14 ZP568s1
cen14 ZP565s1
cen14 378604Xm
cen14 BC187m
cen14 RM11
cen14 YJM981m
cen14 YJM978m
cen14 DBVPG1106m
cen14 ZP579s1
cen14 L1528m
cen14 Y55m
cen14 DBVPG1788m
cen14 DBVPG1373m
cen14 AN3e1s1
cen14 S288cRef
cen14 ARN022As1
cen14 ARN020As1
cen14 ARN019As1
cen14 UWOPS87 2421m
100
cen14 NCYC361m
cen14 322134Sm
cen14 ZP578s1
0.01

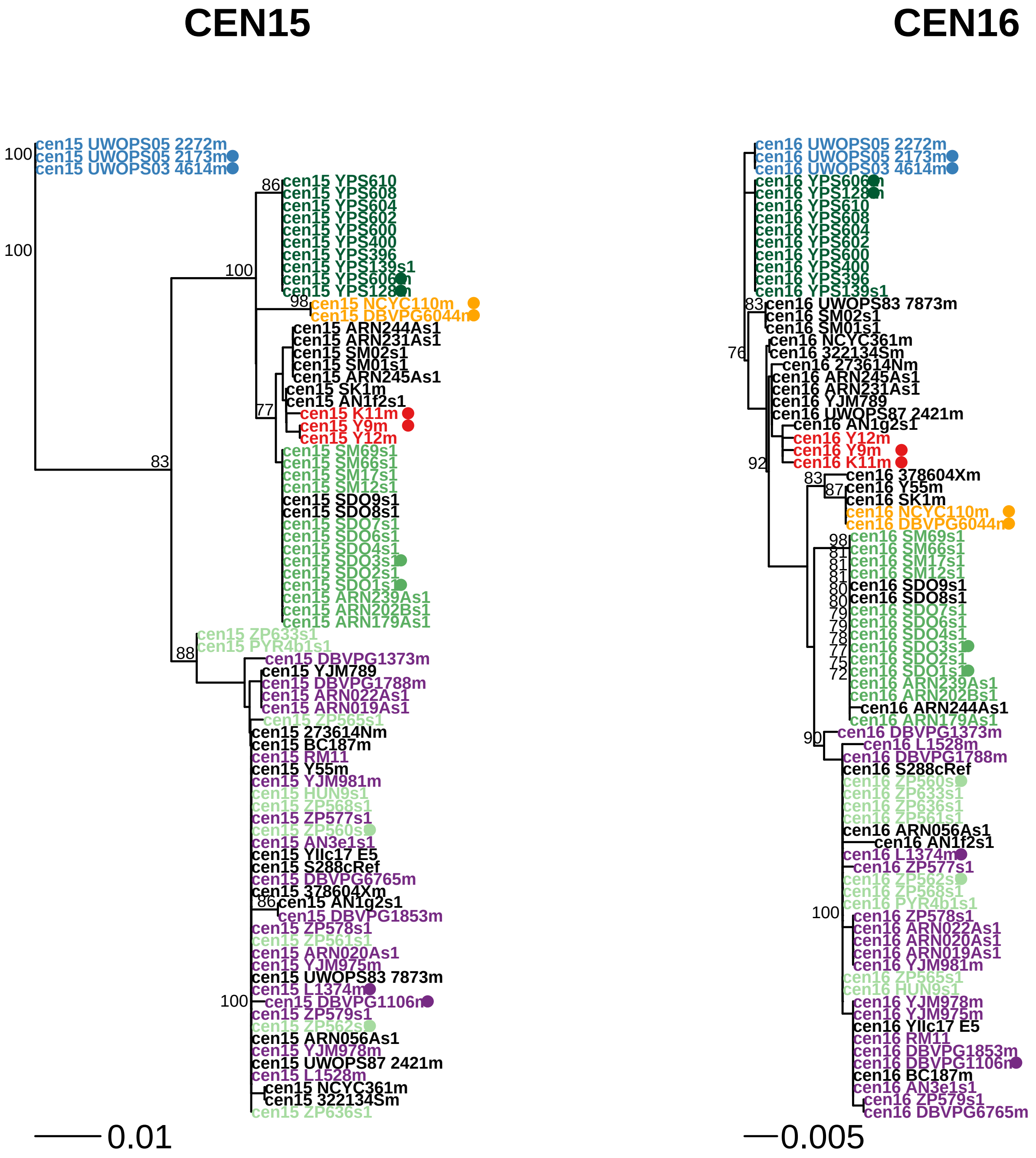

CEN15
CEN16
100
cen15 UWOPS05 2272m
cen15 UWOPS05 2173m
cen15 UWOPS03 4614m
100
86
cen15 YPS610
cen15 YPS608
cen15 YPS604
cen15 YPS602
cen15 YPS600
cen15 YPS400
cen15 YPS396
cen15 YPS139s1
cen15 YPS606m
cen15 YPS128m
100
98
cen15 NCYC110m
cen15 DBVPG6044m
cen15 ARN244As1
cen15 ARN231As1
cen15 SM02s1
cen15 SM01s1
cen15 ARN245As1
cen15 SK1m
cen15 AN1f2s1
77
cen15 K11m
cen15 Y9m
cen15 Y12m
83
cen15 SM69s1
cen15 SM66s1
cen15 SM17s1
cen15 SM12s1
cen15 SDO9s1
cen15 SDO8s1
cen15 SDO7s1
cen15 SDO6s1
cen15 SDO4s1
cen15 SDO3s1
cen15 SDO2s1
cen15 SDO1s1
cen15 ARN239As1
cen15 ARN202Bs1
cen15 ARN179As1
cen15 ZP633s1
cen15 PYR4b1s1
88
cen15 DBVPG1373m
cen15 YJM789
cen15 DBVPG1788m
cen15 ARN022As1
cen15 ARN019As1
cen15 ZP565s1
cen15 273614Nm
cen15 BC187m
cen15 RM11
cen15 Y55m
cen15 YJM981m
cen15 HUN9s1
cen15 ZP568s1
cen15 ZP577s1
cen15 ZP560s1
cen15 AN3e1s1
cen15 YIIc17 E5
cen15 S288cRef
cen15 DBVPG6765m
cen15 378604Xm
86
cen15 AN1g2s1
cen15 DBVPG1853m
cen15 ZP578s1
cen15 ZP561s1
cen15 ARN020As1
cen15 YJM975m
cen15 UWOPS83 7873m
cen15 L1374m
100
cen15 DBVPG1106m
cen15 ZP579s1
cen15 ZP562s1
cen15 ARN056As1
cen15 YJM978m
cen15 UWOPS87 2421m
cen15 L1528m
cen15 NCYC361m
cen15 322134Sm
cen15 ZP636s1
0.01
cen16 UWOPS05 2272m
cen16 UWOPS05 2173m
cen16 UWOPS03 4614m
cen16 YPS606m
cen16 YPS128m
cen16 YPS610
cen16 YPS608
cen16 YPS604
cen16 YPS602
cen16 YPS600
cen16 YPS400
cen16 YPS396
cen16 YPS139s1
83
cen16 UWOPS83 7873m
cen16 SM02s1
cen16 SM01s1
76
cen16 NCYC361m
cen16 322134Sm
cen16 273614Nm
cen16 ARN245As1
cen16 ARN231As1
cen16 YJM789
cen16 UWOPS87 2421m
cen16 AN1g2s1
cen16 Y12m
cen16 Y9m
cen16 K11m
92
83
cen16 378604Xm
87
cen16 Y55m
cen16 SK1m
cen16 NCYC110m
cen16 DBVPG6044m
98
cen16 SM69s1
81
cen16 SM66s1
81
cen16 SM17s1
81
cen16 SM12s1
80
cen16 SDO9s1
80
cen16 SDO8s1
79
cen16 SDO7s1
79
cen16 SDO6s1
78
cen16 SDO4s1
77
cen16 SDO3s1
75
cen16 SDO2s1
72
cen16 SDO1s1
cen16 ARN239As1
cen16 ARN202Bs1
cen16 ARN244As1
cen16 ARN179As1
90
cen16 DBVPG1373m
cen16 L1528m
cen16 DBVPG1788m
cen16 S288cRef
cen16 ZP560s1
cen16 ZP633s1
cen16 ZP636s1
cen16 ZP561s1
cen16 ARN056As1
cen16 AN1f2s1
cen16 L1374m
cen16 ZP577s1
cen16 ZP562s1
cen16 ZP568s1
cen16 PYR4b1s1
100
cen16 ZP578s1
cen16 ARN022As1
cen16 ARN020As1
cen16 ARN019As1
cen16 YJM981m
cen16 ZP565s1
cen16 HUN9s1
cen16 YJM978m
cen16 YJM975m
cen16 YIIc17 E5
cen16 RM11
cen16 DBVPG1853m
cen16 DBVPG1106m
cen16 BC187m
cen16 AN3e1s1
cen16 ZP579s1
cen16 DBVPG6765m
0.005
